# On the Linear Stability of Crystals in the Schrödinger–Poisson Model

**DOI:** 10.1007/s10955-016-1613-x

**Published:** 2016-09-08

**Authors:** A. Komech, E. Kopylova

**Affiliations:** 1 Faculty of Mathematics, Vienna University, Vienna, Austria; 2 Institute for Information Transmission Problems, RAS, Moscow, Russia

**Keywords:** Crystal, Lattice, Ground state, Linear stability, Bloch transform, Hamilton operator, 35L10, 34L25, 47A40, 81U05

## Abstract

We consider the Schrödinger–Poisson–Newton equations for crystals with one ion per cell. We linearize this dynamics at the periodic minimizers of energy per cell and introduce a novel class of the ion charge densities that ensures the stability of the linearized dynamics. Our main result is the *energy positivity* for the Bloch generators of the linearized dynamics under a Wiener-type condition on the ion charge density. We also adopt an additional ‘Jellium’ condition which cancels the negative contribution caused by the electrostatic instability and provides the ‘Jellium’ periodic minimizers and the optimality of the lattice: the energy per cell of the periodic minimizer attains the global minimum among all possible lattices. We show that the energy positivity can fail if the Jellium condition is violated, while the Wiener condition holds. The proof of the energy positivity relies on a novel factorization of the corresponding Hamilton functional. The Bloch generators are nonselfadjoint (and even nonsymmetric) Hamilton operators. We diagonalize these generators using our theory of spectral resolution of the Hamilton operators *with positive definite energy* (Komech and Kopylova in, J Stat Phys 154(1–2):503–521, 2014, J Spectral Theory 5(2):331–361, 2015). The stability of the linearized crystal dynamics is established using this spectral resolution.

## Introduction

Dyson and Lenard [9, 10] were the first to obtain mathematical results on the stability of matter; in their studies a bound from below for the energy was obtained. The thermodynamic limit for the Coulomb systems was first studied by Lebowitz and Lieb [20, 21], see the survey and further development in [22]. These results were extended by Catto, Lions, Le Bris to the Thomas–Fermi and Hartree–Fock models [5–7]. All these results were concerned either with the thermodynamic limit or the existence of a ground state for infinite particle systems. The dynamical stability of ion-electron dynamics for infinite particle systems with moving ions was never examined before. This stability is necessary for a rigorous analysis of fundamental quantum phenomena in the solid state physics: heat conductivity, electric conductivity, thermoelectronic emission, photoelectric effect, Compton effect, etc., see [2].

In present paper, we analyze for the first time the dynamic stability of a crystal periodic minimizer of energy per cell in linear approximation for the simplest Schrödinger–Poisson model. The periodic minimizer for this model was constructed in [16]. The stability for the nonlinear dynamics will be considered elsewhere.

We consider crystals with one ion per cell. The electron cloud is described by the one-particle Schrödinger equation; the ions are looked upon as particles that corresponds to the Born and Oppenheimer approximation. The ions interact with the electron cloud via the scalar potential, which is a solution to the corresponding Poisson equation.

This model does not respect the Pauli exclusion principle for electrons. Nevertheless, it provides a convenient framework to introduce suitable functional tools that might be instrumental for physically more realistic models (the Thomas–Fermi, Hartree–Fock, and second quantized models). In particular, we find a novel stability criterion (1.21), (1.23).

We denote by $\sigma (x)\in L^1({\mathbb R}^3)$ the charge density of one ion,

1.1 $$\begin{aligned} \int _{{\mathbb R}^3} \sigma (x)dx=eZ>0, \end{aligned}$$

where $e>0$ is the elementary charge. We assume througout the paper that

1.2 $$\begin{aligned} \langle x\rangle ^4\sigma \in L^2({\mathbb R}^3),\qquad (\Delta -1)\sigma \in L^1({\mathbb R}^3). \end{aligned}$$

We consider the cubic lattice $\Gamma = {\mathbb Z}^3$ for the simplicity of notations. Let $\psi (x,t)$ be the wave function of the electron field, *q*(*n*, *t*) denote the ions displacements, and $\Phi (x)$ be the electrostatic potential generated by the ions and electrons. We assume that $\hbar =c=\mathrm{m}=1$, where *c* is the speed of light and $\mathrm{m}$ is the electron mass. The coupled Schrödinger–Poisson–Newton equations read

1.3 $$\begin{aligned} i\dot{\psi }(x,t)= & {} -\frac{1}{2}\Delta \psi (x,t)-e\Phi (x,t)\psi (x,t),\qquad x\in {\mathbb R}^3, \end{aligned}$$

1.4 $$\begin{aligned} -\Delta \Phi (x,t)= & {} \rho (x,t):=\sum _n\sigma (x-n-q(n,t))-e|\psi (x,t)|^2,\qquad x\in {\mathbb R}^3, \end{aligned}$$

1.5 $$\begin{aligned} M\ddot{q}(n,t)= & {} -\langle \mathbf{\nabla }\Phi (x,t),\sigma (x-n-q(n,t))\rangle , \qquad n\in {\mathbb Z}^3. \end{aligned}$$

Here the brackets stand for the Hermitian scalar product in the Hilbert space $L^2({\mathbb R}^3)$ and for its various extensions, the series (1.4) converges in a suitable sense, and $M>0$. All the derivatives here and below are understood in the sense of distributions. These equations can be written as a Hamilton system with formal Hamilton functional

1.6 $$\begin{aligned} {\mathscr {H}}(\psi ,q,p)=\frac{1}{2}\int _{{\mathbb R}^3}[|\nabla \psi (x)|^2+\rho (x)G\rho (x)]dx+\sum _{n} \frac{p^2(n)}{2M}, \end{aligned}$$

where $q:=(q(n): ~n\in {\mathbb Z}^3)$, $p:=(p(n): ~n\in {\mathbb Z}^3)$, $\rho (x)$ is defined similarly to (1.4), and $G:=(-\Delta )^{-1}$, i.e.,

1.7 $$\begin{aligned} G\rho (x):=\displaystyle \frac{1}{4\pi }\int \frac{\rho (y)dy}{|x-y|},\qquad x\in {\mathbb R}^3. \end{aligned}$$

Namely, the system (1.3)–(1.5) can be formally written as

1.8 $$\begin{aligned} i\dot{\psi }(x,t)=\partial _{\overline{\psi }(x)}{\mathscr {H}}, ~~~ \dot{q}(n,t)=\partial _{p(n)}{\mathscr {H}}, ~~~ \dot{p}(n,t)=-\!\partial _{q(n)}{\mathscr {H}}, \end{aligned}$$

where $\partial _{\overline{z}}:=\frac{1}{2}(\partial _{z_1}+i\partial _{z_2})$ with $z_1=\mathrm{Re{\,}}z$ and $z_2=\mathrm{Im{\,}}z$.

We investigate the stability of periodic minimizers of the energy per cell, which are $\Gamma$-periodic stationary solutions of (1.3)–(1.5). We will see that these periodic minimizers can be stable or unstable (then the true ground state of the system might be non-periodic, e.g., quasiperiodic), depending on the choice of the nuclear density $\sigma$. However, we only study very special densities $\sigma$ satisfying some conditions discussed below. A periodic minimizer of a crystal is a $\Gamma$-periodic stationary solution

1.9 $$\begin{aligned} \psi ^0(x)e^{-i\omega ^0 t}~,~~~ \Phi ^0(x)~,~~~~q^0(n)=q^0~~\mathrm{and}~~p^0(n)=0~~~~ \mathrm{for}~~ n\in {\mathbb Z}^3 \end{aligned}$$

with a real $\omega ^0$. Such periodic minimizer was constructed in [16] for general lattice with several ions per cell. In our case the ion position $q^0\in {\mathbb R}^3$ can be chosen arbitrarily, and we set $q^0=0$ everywhere below.

In present paper, we prove the stability of the *formal linearization* of the nonlinear system (1.3)–(1.5) at the periodic minimizer (1.9). Namely, substituting

1.10 $$\begin{aligned} \psi (x,t)=[\psi ^0(x)+\Psi (x,t)]e^{-i\omega ^0 t} \end{aligned}$$

into the nonlinear equations (1.3), (1.5) with $\Phi (x,t)=G\rho (x,t)$, we *formally* obtain the linearized equations (see Appendix 1)

1.11 $$\begin{aligned} \left. \begin{array}{rcl} &{}&{} [i\partial _t+\omega ^0]\Psi (x,t)=-\frac{1}{2}\Delta \Psi (x,t)-e\Phi ^0(x)\Psi (x,t)-e\psi ^0(x)G\rho _1(x,t)\\ &{}&{}\dot{q}(n,t)=p(n,t)/M\\ &{}&{} \dot{p}(n,t)=-\langle \mathbf{\nabla }G\rho _1(t), \sigma (x-n)\rangle +\langle \nabla \Phi ^0, q(n,t)\cdot \nabla \sigma (x-n) \rangle \end{array}\right| \begin{array}{c} x\in {\mathbb R}^3\\ n\in {\mathbb Z}^3 \end{array}\nonumber \\ \end{aligned}$$

Here $\rho _1(x,t)$ is the linearized charge density

1.12 $$\begin{aligned} \rho _1(x,t)=-\sum _n q(n,t)\cdot \nabla \sigma (x-n) -2e\mathrm{Re{\,}}[\psi ^0(x)\overline{\Psi (x,t)}]. \end{aligned}$$

The system (1.11) is linear over ${\mathbb R}$, but it is not complex linear. This is due to the last term in (1.12), which appears from the linearization of the term $|\psi |^2=\psi \overline{\psi }$ in (1.4). However, we need the complex linearity for the application of the spectral theory. That is why we will consider below the complexification of system (1.11) by writing it in the variables $\Psi _1(x,t):=\mathrm{Re{\,}}\Psi (x,t),\Psi _2(x,t):=\mathrm{Im{\,}}\Psi (x,t)$.

The periodic minimizer $\psi ^0(x)$ is a real function up to a phase factor $e^{i\phi }$ (see [1] and (1.24) below). This factor can be canceled by multiplying $\psi ^0(x)$ and $\Psi (x,t)$ by $e^{-i\phi }$ in the first equation (1.11) and in (1.12). Therefore, we will assume that $\psi ^0(x)$ is a real function, and hence,

1.13 $$\begin{aligned} \mathrm{Re{\,}}[\psi ^0(x)\overline{\Psi (x,t)}]=\psi ^0(x)\Psi _1(x,t). \end{aligned}$$

Then (1.11) can be written as

1.14 $$\begin{aligned} \dot{Y}(t)=AY(t),\qquad A=\left( \begin{array}{ccrl} 0 &{}\quad H^0 &{}\quad 0 &{}\quad 0\\ -H^0-2e^2\psi ^0G\psi ^0 &{}\quad 0 &{}\quad -S &{}\quad 0\\ 0 &{}\quad 0 &{}\quad 0 &{}\quad M^{-1}\\ -2S^{{\,}*} &{}\quad 0 &{}\quad -T &{}\quad 0\\ \end{array}\right) , \end{aligned}$$

where we denote $Y(t)=(\Psi _1(\cdot ,t),\Psi _2(\cdot ,t),q(\cdot ,t),p(\cdot ,t))$, $H^0:=-\frac{1}{2}\Delta -e\Phi ^0(x)-\omega ^0$, the operators *S* and *T* correspond to matrices (3.3) and (3.4), respectively, and $\psi ^0$ denotes the operators of multiplication by the real function $\psi ^0(x)$. The Hamilton representation (1.8) implies that

1.15 $$\begin{aligned} A= & {} JB,\qquad B= \left( \begin{array}{cccl} 2H^0+4e^2\psi ^0 G\psi ^0 &{}\quad 0 &{}\quad 2S &{}\quad 0 \\ 0 &{}\quad 2H^0 &{}\quad 0 &{}\quad 0\\ 2S^{{\,}*} &{}\quad 0 &{}\quad T &{}\quad 0 \\ 0 &{}\quad 0 &{}\quad 0 &{}\quad M^{-1} \\ \end{array}\right) ,\nonumber \\ J= & {} \left( \begin{array}{cccc} 0 &{}\quad \frac{1}{2} &{}\quad 0 &{}\quad 0\\ -\frac{1}{2} &{}\quad 0 &{}\quad 0 &{}\quad 0\\ 0 &{}\quad 0 &{}\quad 0 &{}\quad 1\\ 0 &{}\quad 0 &{}\quad -1 &{}\quad 0 \end{array}\right) . \end{aligned}$$

Our main result is the stability of the linearized system (1.14): for any initial state of finite energy there exists a unique global solution which is bounded in the energy norm.

We show that the generator *A* is densely defined in the Hilbert space

1.16 $$\begin{aligned} {{\mathscr {Y}}^0}:=L^2({\mathbb R}^3)\oplus L^2({\mathbb R}^3)\oplus {\mathbb R}^3\oplus {\mathbb R}^3 \end{aligned}$$

and commutes with translations by vectors from $\Gamma$. Hence, the equation (1.14) can be reduced with the help of the Fourier–Bloch–Gelfand–Zak transform to equations with the corresponding Bloch generators $\tilde{A}(\theta )=J\tilde{B}(\theta )$, which depend on the parameter $\theta$ from the Brillouin zone $\Pi ^*:=[0,2\pi ]^3$. The Bloch energy operator $\tilde{B}(\theta )$ is given by

1.17 $$\begin{aligned} \tilde{B}(\theta ) =\!\left( \!\begin{array}{cccl} 2\tilde{H}^0(\theta )+4e^2\psi ^0 \tilde{G}(\theta )\psi ^0&{}\quad 0 &{}\quad 2\tilde{S}(\theta )&{}\quad 0 \\ 0 &{}\quad 2\tilde{H}^0(\theta )&{}\quad 0 &{}\quad 0 \\ 2\tilde{S}^{{\,}*}(\theta ) &{}\quad 0 &{}\quad \hat{T}(\theta ) &{}\quad 0 \\ 0 &{}\quad 0 &{}\quad 0 &{}\quad M^{-1} \\ \end{array}\!\right) ,\qquad \theta \in \Pi ^* \setminus \Gamma ^*,\nonumber \\ \end{aligned}$$

where $\Gamma ^*:=2\pi {\mathbb Z}^3$, and $\tilde{H}^0(\theta ):=-\frac{1}{2}(\nabla -i\theta )^2-e\Phi ^0(x)-\omega ^0$. Further, $\tilde{G}(\theta )$ is the inverse of the operator $(i\nabla +\theta )^2:H^2(T^3)\rightarrow L^2(T^3)$. Finally, $\tilde{S}(\theta )$ and $\hat{T}(\theta )= \hat{T}_2(\theta )+\hat{T}_1(\theta )$ are defined, respectively, by (6.22) and (3.9), (3.12).

The operator $\tilde{B}(\theta )$ is selfadjoint in the Hilbert space ${{\mathscr {Y}}^0}(T^3)$ with the domain ${{\mathscr {Y}}^2}(T^3)$, where we denote

1.18 $$\begin{aligned} {{\mathscr {Y}}^s}(T^3):=H^s(T^3)\oplus H^s(T^3)\oplus {\mathbb C}^3\oplus {\mathbb C}^3,\qquad T^3:={\mathbb R}^3/\Gamma \end{aligned}$$

for $s\in {\mathbb R}$; its spectrum is discrete. However, the operator *A* is not selfadjoint and even not symmetric in ${\mathscr {Y}}^0$ – this a typical situation in the linearization of *U*(1)-invariant nonlinear equations [17, Appendix B]. Respectively, the Bloch generators $\tilde{A}(\theta )$ are not selfadjoint in ${{\mathscr {Y}}^0}(T^3)$

The main crux here is that we cannot apply the von Neumann spectral theorem to the nonselfadjoint generators *A* and $\tilde{A}(\theta )$. We solve this problem by applying our spectral theory of abstract Hamilton operators with positive energy [17, 18]. This is why we need the positivity of the energy operator $\tilde{B}(\theta )$: for $\tilde{Y}\in {{\mathscr {Y}}^2}(T^3)$

1.19 $$\begin{aligned} {\mathscr {E}}(\theta ,\tilde{Y}):=\langle \tilde{Y}, \tilde{B}(\theta )\tilde{Y}\rangle _{{{\mathscr {Y}}^0}(T^3)}\ge \varkappa (\theta )\Vert \tilde{Y}\Vert _{{{\mathscr {Y}}^1}(T^3)}^2, \quad \mathrm{where }\,\, \varkappa (\theta )>0\,\,\,\, \mathrm{for\; a.e.}~~\theta \in \Pi ^*\setminus \Gamma ^* \nonumber \\ \end{aligned}$$

and the brackets denote the scalar product in ${{\mathscr {Y}}^0}(T^3)$. Equivalently,

1.20 $$\begin{aligned} \tilde{B}_0(\theta ):=\inf \,\,\mathop {\mathrm {Spec}\,}\nolimits \tilde{B}(\theta )>0\qquad \mathrm{for\, a.e.}~~\theta \in \Pi ^*\setminus \Gamma ^*. \end{aligned}$$

The main result of the present paper is the proof of the positivity (1.20) for the ions charge densities $\sigma$ satisfying the following two conditions C1 and C2 on the corresponding Fourier transform $\tilde{\sigma }(\xi )$.1.21where the series converges by (1.2). Equivalently,

1.22 $$\begin{aligned} \Sigma _0(\theta )>0 \qquad \quad ~~\mathrm{for\ a.e.}~~\theta \in \Pi ^*\setminus \Gamma ^*, \end{aligned}$$

where $\Sigma _0(\theta )$ is the minimal eigenvalue of the matrix $\Sigma (\theta )$. This condition is an analog of the Fermi Golden Rule for crystals.1.23This condition immediately implies that the periodized ions charge density corresponding to the periodic minimizer is a positive constant everywhere in space. In this case the minimum of energy per cell corresponds to the opposite uniform negative electronic charge, so these ion and electronic densities cancel each other, and the potential $\Phi (x,t)$ vanishes by (1.4),

1.24 $$\begin{aligned} \psi _0(x)\equiv e^{i\phi }\sqrt{Z},\quad \phi \in [0, 2\pi ];\quad \Phi _0(x)\equiv 0,\quad \omega _0=0. \end{aligned}$$

The energy per cell attains its minimum since the integral (2.8) vanishes (see Lemma 2.1).

Thus, the condition (1.23) means that ions can be arranged on an appropriate lattice in a way that their total charge density is constant everywhere in space. This clearly requires that $\sigma$ has the symmetry of this lattice, which is false for radial densities. The simplest example of such a $\sigma$ is a constant over the unit cell of a given lattice, which is what physicists usually call Jellium [11]. Here we study this model in the rigorous context of the Schrödinger-Poisson equations. The outstanding role in this Jellium model in our context is provided by the optimality of the lattice $\Gamma$: under the condition (1.23) the energy of the periodic minimizer per cell attains the global minimum among all possible lattices (see Lemmas 2.1 and 2.2).

We prove that the stability of this constant-density state under small deformations, is equivalent to the simple condition (1.21). In that case this Jellium periodic minimizer is the crystal ground state, i.e., its small local deformations have a higher energy as well as other periodic arrangements. Also, we use the positivity (1.20) to give a meaning to the associated linearized dynamics, using existing results [17, 18].

It is to be noticed that (1.21) is satisfied for the simplest Jellium model, when $\sigma$ is constant in the unit cell: in this case the Fourier tranform $\tilde{\sigma }$ is the ‘Dirichlet kernel’. Actually, the condition (1.21) holds “generically”.

We prove (1.20) with

1.25 $$\begin{aligned} \tilde{B}_0(\theta )\ge \varepsilon d^4(\theta )\Sigma _0(\theta ),\qquad \theta \in \Pi ^*\setminus \Gamma ^*, \end{aligned}$$

where $\varepsilon >0$ is sufficiently small and $d(\theta ):=\mathrm dist{\,}(\theta ,\Gamma ^*)$. This implies that $\mathop {\mathrm {Spec}\,}\nolimits B\subset [0,\infty )$. Moreover, we show in Theorem 7.3 (ii) that

1.26 $$\begin{aligned} \tilde{B}_0(\theta )\le \Sigma _0(\theta ),\qquad \theta \in \Pi ^*\setminus \Gamma ^*. \end{aligned}$$

This inequality implies that $0\in \mathop {\mathrm {Spec}\,}\nolimits B$. Indeed, the conditions (1.21) and (1.23) imply that $\Sigma (\theta )$ is a continuous $\Gamma ^*$-periodic function, which admits the asymptotics

1.27 $$\begin{aligned} \Sigma (\theta )\sim \frac{\theta \otimes \theta }{|\theta |^2}\tilde{\sigma }(0)+{\mathscr {O}}(|\theta |^2),\qquad \theta \rightarrow 0. \end{aligned}$$

However, the matrix $\theta \otimes \theta$ is degenerate, and hence, $\Sigma _0(\theta )\rightarrow 0$ as $\theta \rightarrow 0$ by the asymptotics (1.27). Therefore, the positivity (1.20) breaks down at $\theta \in \Gamma ^*\cap \Pi ^*$ by (1.26). Examples 7.1 and 7.2 demonstrate that the positivity can also break down at some other points and submanifolds of $\Pi ^*$ that depend on the ion charge density $\sigma$.

Let us comment on our approach. The structure of the periodic minimizer (1.24) under condition (1.23) seems trivial. However, even in this case the proof of the positivity (1.20) is not straightforward, since the operators $\tilde{S}(\theta )$ and $\hat{T}(\theta )$ in $\tilde{B}(\theta )$ depend on the fuctional parameter $\sigma$. Our proof of (1.20) relies on (i) a novel factorization (7.8) of the matrix elements of $\tilde{B}(\theta )$, and (ii) Sylvester-type arguments for matrix operators (see Remark 7.6).

We show that the condition (1.21) is necessary for the positivity (1.20). We expect that the condition (1.23) is also necessary for the positivity (1.20), however, this is still an open challenging problem. This condition cancels the negative energy which is provided by the electrostatic instability (‘Earnshaw’s Theorem’ [29], see Remark 10.2). At least we show in Lemma 10.1 that the positivity (1.20) can break down when condition (1.23) fails. This counterexample relies on a novel small-charge asymptotics of the periodic minimizer$\psi ^0(x)$ (Lemma 9.1).

Finally, the positivity (1.20) allows us to construct the spectral resolution of $\tilde{A}(\theta )$, which results in the stability for the linearized dynamics (1.14). The spectral resolution is constructed with application of our spectral theory of abstract Hamilton operators [17, 18]. This theory is an infinite-dimensional version of some Gohberg and Krein ideas from the theory of parametric resonance [14, Chap. VI].

In concluzion, all our methods and results extend obviously to equations (1.3)–(1.5) in the case of general lattice

1.28 $$\begin{aligned} \Gamma =\{n_1a_1+n_2a_2+n_3a_3: (n_1,n_2,n_3)\in {\mathbb Z}^3\}, \end{aligned}$$

where the generators $a_k\in {\mathbb R}^3$ are linearly independent. In this case the condition (1.23) becomes

1.29 $$\begin{aligned} \tilde{\sigma }(\gamma ^*)=0,\quad \gamma ^*\in \Gamma ^*\setminus 0, \end{aligned}$$

where $\Gamma ^*$ denotes the dual lattice, i.e., $\Gamma ^*=\{m_1b_1+m_2b_2+m_3b_3: (m_1,m_2,m_3)\in {\mathbb Z}^3\}$ with $\langle a_k,b_j\rangle =2\pi \delta _{kj}$. The condition (1.29) claryfies the relation between the properties of the ions and the resulting crystal geometry.

### Remark 1.1

Conditions (1.23), (1.29) seem to be rather restrictive. On the other hand, the distinction between the ions and electron field is not too sharp, since each ion contains in itself a number of bonding electrons. Physically, the ion charge density $\sigma (x)$ might vary during the process of the crystal formation due to interaction with the electron field. Respectively, one could expect that identities (1.23), (1.29) may result from this process.

Our main novelties are as follows:

I. The energy positivity (1.20) under conditions (1.21) and (1.23).
II. Spectral resolution of nonselfadjoint Hamilton generators and stability of the linearized dynamics.
III. An asymptotics of the periodic minimizer as $e\rightarrow 0$.
IV. An example of negative energy when the condition (1.23) breaks down.
V. The optimality of the lattice $\Gamma$ under conditions (1.23), (1.29).

Let us comment on previous results in these directions.

The crystal periodic minimizer for the Hartree–Fock equations was constructed by Catto, Le Bris, and Lions [6, 7]. For the Thomas–Fermi model similar results were obtained in [5].

The corresponding periodic minimizer in the Schrödinger–Poisson model was constructed in [16]. The stability for the linearized dynamics was not established previously in any model.

In [4], Cancès and Stoltz have established the well-posedness for local perturbations of the stationary density matrix in an infinite crystal for the reduced Hartree–Fock model in the *random phase approximation* with the Coulomb pairwise interaction potential $w(x-y)=1/|x-y|$. The space-periodic nuclear potential in the equation (3) of [4] does not depend on time, which corresponds to fixed ion positions.

The nonlinear Hartree–Fock dynamics with the Coulomb potential without the random phase approximation was not previously examined, see the discussion in [19] and in the introductions of the papers [3, 4].

The paper [3] deals with random reduced HF model of crystal when the ions charge density and the electron density matrix are random processes and the action of the lattice translations on the probability space is ergodic. The authors obtain suitable generalizations of the Hoffmann–Ostenhof and Lieb–Thirring inequalities for ergodic density matrices and construct random potentials which are solutions to the Poisson equation with the corresponding stationary stochastic charge density. The main result is the coincidence of this model with the thermodynamic limit in the case of the short-range Yukawa interaction.

In [23], Lewin and Sabin established the well-posedness for the reduced von Neumann equation with density matrices of infinite trace, describing the Fermi gas with pair-wise interaction potentials $w\in L^1({\mathbb R}^3)$. They also proved the asymptotic stability of stationary states for 2D Fermi gas [24].

Traditional *one-electron* Bethe–Bloch–Sommerfeld mathematical model of crystals reduces to the linear Schrödinger equation with a space-periodic static potential, which corresponds to the standing ions. The corresponding spectral theory is well developed, see [27] and the references therein. The scattering theory for short-range and long-range perturbations of such ‘periodic operators’ was constructed in [12, 13].

The paper is organized as follows. In Sect. 2 we recall our result [16] on the existence of a periodic minimizer In Sects. 3–5 we study the Hamiltonian structure of the linearized dynamics and find a bound of the energy from below. In Sect. 6 we calculate the generator of the linearized dynamics in the Fourier–Bloch representation. In Sect. 7 we prove the positivity of the energy. In Sect. 8 we apply this positivity to the stability of the linearized dynamics. Finally, in Sects. 9 and 10 we establish small charge asymptotics of the periodic minimizer and construct examples of negative energy. Some technical calculations are carried out in Appendices.

## Space-Periodic Minimizers

Let us recall the results of [16] on the existence of the periodic minimizer (1.9). Substituting (1.9) with $q^0=0$ into (1.3)–(1.5), we obtain the system

2.1 $$\begin{aligned} \omega ^0\psi ^0(x)= & {} -\frac{1}{2}\Delta \psi ^0(x)-e\Phi ^0(x)\psi ^0(x),\qquad x\in T^3:={\mathbb R}^3/\Gamma , \end{aligned}$$

2.2 $$\begin{aligned} -\Delta \Phi ^0(x)= & {} \rho ^0(x):=\sigma ^0(x)-e|\psi ^0(x)|^2,\qquad x\in T^3, \end{aligned}$$

2.3 $$\begin{aligned} 0= & {} - {\,}\langle \mathbf{\nabla }\Phi ^0(x), \sigma (x-n)\rangle ,\qquad n\in {\mathbb Z}^3, \end{aligned}$$

where we denote the corresponding periodized ion charge density

2.4 $$\begin{aligned} \sigma ^0(x):= \sum _{n}\sigma (x-n). \end{aligned}$$

The Poisson equation (2.2) for the $\Gamma$-periodic potential $\Phi ^0$ implies the neutrality of the periodic cell $T^3={\mathbb R}^3/\Gamma$,

2.5 $$\begin{aligned} \int _{T^3} \rho ^0(x)dx=0, \end{aligned}$$

which is equivalent to the normalization condition

2.6 $$\begin{aligned} \int _{T^3} |\psi ^0(x)|^2\,dx=Z \end{aligned}$$

by (1.1). We assume that $$ Z>0$$, since otherwise the theory is trivial.

### The Regularity of the Periodic Minimizer

The existence of the periodic minimizer (1.9) is proved in [16] under the condition

2.7 $$\begin{aligned} \sigma ^0\in L^2(T^3) \end{aligned}$$

which holds by (1.2). The periodic minimizer $\psi ^0$ is constructed as a minimal point of the energy per cell

2.8 $$\begin{aligned} U(\psi )=\frac{1}{2}\int _{T^3}[|\nabla \psi (x)|^2+\rho (x)G_\mathrm{per}\rho (x)]dx, \end{aligned}$$

where

2.9 $$\begin{aligned} \rho (x):=\sigma ^0(x)-e|\psi (x)|^2, \end{aligned}$$

while the operator $G_\mathrm{per}$ is defined by

2.10 $$\begin{aligned} G_\mathrm{per}\varphi (x)=\sum _{m\in {\mathbb Z}^3\setminus 0}e^{-i2\pi mx}\frac{\check{\varphi }(m)}{|2\pi m|^2}, \qquad \check{\varphi }(m)=\int _{T^3} e^{i2\pi mx}\varphi (x)dx. \end{aligned}$$

More precisely,

2.11 $$\begin{aligned} U(\psi ^0)=\min _{\psi \in {\mathscr {M}}} U(\psi ), \end{aligned}$$

where ${\mathscr {M}}$ denotes the manifold

2.12 $$\begin{aligned} {\mathscr {M}}:=\{ \psi \in H^1(T^3):\int _{T^3}|\psi (x)|^2\,dx=Z\}. \end{aligned}$$

The results [16] imply that there exists a periodic minimizer with $\psi ^0,\Phi ^0\in H^2(T^3)$. Hence $\psi ^0\Phi ^0\in H^2(T^3)$, and the Eq. (2.1) implies that

2.13 $$\begin{aligned} \psi ^0\in H^4(T^3)\subset C^2_b(T^3). \end{aligned}$$

In other words,

2.14 $$\begin{aligned} \psi ^0(x)=\sum _{m\in {\mathbb Z}^3} \check{\psi }^0(m)e^{i2\pi m x},\qquad \sum _{m\in {\mathbb Z}^3} \langle m\rangle ^8|\check{\psi }^0(m)|^2<\infty ,\qquad \langle m\rangle :=(1+|m|^2)^{1/2}.\nonumber \\ \end{aligned}$$

### The ‘Jellium periodic minimizer’ and Optimality of the Lattice

The following lemma means that under the condition (1.23) the energy of the periodic minimizer per cell attains at $\Gamma$ the *global minimum* among all possible lattices.

#### Lemma 2.1

Let the ion density $\sigma (x)$ satisfy (2.7) and (1.23). Then formulas (1.24) give the set of all minimizers of energy per cell (2.8), and the corresponding energy per cell is zero.

#### Proof

First we note that

2.15 $$\begin{aligned} \tilde{\sigma }(0)=\int \sigma (x)dx=eZ>0 \end{aligned}$$

by (1.1). Hence, the corresponding periodized ion charge density equals $\sigma ^0(x):=\sum \sigma (x-n)\equiv eZ$, since its Fourier coefficients with nonzero numbers vanish by (1.23):

2.16 $$\begin{aligned} \check{\sigma }^0(m)=\int _{T^3} e^{i2\pi m x}\sigma ^0(x)dx= \int _{R^3} e^{i2\pi m x}\sigma (x)dx=\tilde{\sigma }(2\pi m)= 0,\qquad m\in {\mathbb Z}^3\setminus 0.\nonumber \\ \end{aligned}$$

Therefore, functions (1.24) give a solution to (2.1)–(2.3) with zero energy per cell (2.8). On the other hand, the energy (2.8) is nonnegative, and it is zero only for functions (1.24).$\square$

We can also consider equations (2.1)–(2.4) in the case of a general lattice (1.28). The following lemma gives a simple test for the energy of the periodic minimizer per cell attains at $\Gamma ={\mathbb Z}^3$ the *strong local minimum* among all possible lattices.

#### Lemma 2.2

Let the conditions (2.7) and (1.23) hold and $\Gamma ={\mathbb Z}^3$. Let the Wiener condition (1.21) hold for **each** $\theta \in \Pi ^*\setminus \Gamma ^*$. Then for any lattice $\Gamma _1\not \subset \Gamma$, the energy per cell (2.11) is strictly positive.

#### Proof

Let $\psi ^0_1$ denote a periodic minimizer for the lattice $\Gamma _1$. There exists at least one point $\gamma _1\in \Gamma _1^*\setminus \Gamma ^*$. Hence $\tilde{\sigma }(\gamma _1)\ne 0$ by (1.21) with each $\theta \in \Pi ^*\setminus \Gamma ^*$. This means that at least one of the Fourier coefficients (2.16), with $\gamma _1$ instead of $2\pi m$, does not vanish. Therefore, the corresponding periodized ion charge density

2.17 $$\begin{aligned} \sigma ^0_1(x)\not \equiv \mathop {\mathrm {const}}\nolimits ,\qquad x\in {\mathbb R}^3. \end{aligned}$$

This implies that

2.18 $$\begin{aligned} \psi ^0_1(x)\not \equiv \mathop {\mathrm {const}}\nolimits ,\qquad x\in {\mathbb R}^3. \end{aligned}$$

Indeed, the equation (2.1) with $\psi ^0_1(x)\equiv \mathop {\mathrm {const}}\nolimits \ne 0$ would imply that $\omega ^0\equiv -e\Phi ^0_1(x)$. Then the Poisson equation (2.2) gives $\sigma ^0_1(x)-e|\psi ^0_1(x)|^2\equiv 0$, which contradicts (2.17). Finally, (2.18) implies that the energy per cell (2.8) for $\psi ^0_1$ is strictly positive. $\square$

## Linearized Dynamics

Let us calculate the entries of the matrix operator (1.14) under conditions (1.2). For $f(x)\in C_0^\infty ({\mathbb R}^3)$ the Fourier transform is defined by

3.1 $$\begin{aligned} f(x)=\frac{1}{(2\pi )^3}\int _{{\mathbb R}^3} e^{-i\xi x}\tilde{f}(\xi )d\xi ,\qquad x\in {\mathbb R}^3; \qquad \tilde{f}(\xi )=\int _{{\mathbb R}^3} e^{i\xi x}f(x)dx,\qquad \xi \in {\mathbb R}^3.\nonumber \\ \end{aligned}$$

The conditions (1.2) imply that

3.2 $$\begin{aligned} (\Delta -1)\tilde{\sigma }\in L^2({\mathbb R}^3),\qquad \langle \xi \rangle ^2\tilde{\sigma }(\xi )\le \mathop {\mathrm {const}}\nolimits . \end{aligned}$$

Let us recall that the periodic minimizer $\psi ^0(x)$ can be taken to be a real function. In this case (1.11)–(1.13) imply that the operator-matrix *A* is given by (1.14), where *S* denotes the operator with the ‘matrix’

3.3 $$\begin{aligned} S(x,n):=e\psi ^0(x)G\nabla \sigma (x-n):~~n\in {\mathbb Z}^3,~x\in {\mathbb R}^3. \end{aligned}$$

Finally, *T* is the real matrix with entries

3.4 $$\begin{aligned} T(n,n'):= & {} -\displaystyle \langle G\nabla \otimes \nabla \sigma (x-n'), \sigma (x-n) \rangle + \displaystyle \langle \Phi ^0,\nabla \otimes \nabla \sigma \rangle \delta _{nn'}\nonumber \\= & {} T_1(n-n')+T_2(n-n'). \end{aligned}$$

The operators $G\psi ^0: L^2({\mathbb R}^3)\rightarrow L^2({\mathbb R}^3)$ and $S:l^2:=l^2({\mathbb Z}^3)\otimes {\mathbb C}^3\rightarrow L^2({\mathbb R}^3)$ are not bounded due to the ‘infrared divergence’, see Remark 4.4. In the next section, we will construct a dense domain for all these operators.

On the other hand, the corresponding operators $T_1$ and $T_2$ are bounded in view of the following lemma. Denote by $\Pi$ the primitive cell

3.5 $$\begin{aligned} \Pi :=\{(x_1,x_2,x_3):0\le x_k\le 1,~k=1,2,3\}. \end{aligned}$$

Let us define the Fourier transform on $l^2$ as

3.6 $$\begin{aligned} \hat{q}(\theta )=\sum _{n\in {\mathbb Z}^3} e^{in\theta }q(n)\qquad ~~\mathrm{for\ a.e.}~~ \theta \in \Pi ^*;\qquad q(n)=\frac{1}{|\Pi ^*|}\int _{\Pi ^*} e^{-in\theta }\hat{q}(\theta )d\theta ,~~n\in {\mathbb Z}^3,\nonumber \\ \end{aligned}$$

where $\Pi ^*=2\pi \Pi$ denotes the primitive cell of the lattice $\Gamma ^*$, the series converging in $L^2(\Pi ^*)$.

### Lemma 3.1

Let conditions (1.2) and (2.13) hold. Then

(i) The operators $T_1$ and $T_2$ are bounded in $l^2$.
(ii) $T_2=0$ under condition (1.23).

### Proof

The first operator $T_1$ reads as the convolution $T_1 q(n)=\sum T_1(n-n')q(n')$, where

3.7 $$\begin{aligned} T_1(n)=-\langle G\nabla \otimes \nabla \sigma (x), \sigma (x-n) \rangle . \end{aligned}$$

By the Fourier transform (3.6), the convolution operator $T_1$ becomes the multiplication,

3.8 $$\begin{aligned} \widehat{T_1 q}(\theta )=\hat{T}_1(\theta ) \hat{q}(\theta )\qquad ~~\mathrm{for\ a.e.}~~\theta \in \Pi ^*\setminus \Gamma ^*. \end{aligned}$$

By the Bessel-Parseval identity it suffices to check that the ‘symbol’ $\hat{T}_1(\theta )$ is a bounded function. This follows by direct calculation from (3.4). First, we apply the Parseval identity3.9since the last sum over *n* equals $\displaystyle |\Pi ^*|\sum \nolimits _m \delta (\theta +\xi -2\pi m)$ by the Poisson summation formula [15]. Finally, $|\tilde{\sigma }(\xi )|\le C\langle \xi \rangle ^{-2}$ by (3.2). Hence,

3.10 $$\begin{aligned} \Vert \hat{T}_1(\theta )\Vert \le \sum _m| \tilde{\sigma }(2\pi m-\theta )|^2\le C^2\sum _m\langle m\rangle ^{-4}<\infty . \end{aligned}$$

Finally,

3.11 $$\begin{aligned} \widehat{T_2q}(\theta )=\hat{T}_2\hat{q}(\theta ),\qquad \theta \in \Pi ^*, \end{aligned}$$

where

3.12 $$\begin{aligned} \hat{T}_2=\langle \Phi ^0(x), \nabla \otimes \nabla \sigma (x)\rangle . \end{aligned}$$

The matrix is finite by (1.2 ), since $\Phi ^0\in H^2(T^3)$ is a bounded periodic function.

(ii) (3.12) and (1.24) imply that $T_2=0$ under condition (1.23).$\square$

## The Hamilton Structure and the Domain

In this section we study the domain of the generator *A* given by (1.14) and (1.15).

### Definition 4.1

(i) ${\mathscr {S}}_+:= \cup _{\varepsilon >0} {\mathscr {S}}_\varepsilon$, where ${\mathscr {S}}_\varepsilon$ is the space of functions $\Psi \in {\mathscr {S}}({\mathbb R}^3)$ whose Fourier transforms $\hat{\Psi }(\xi )$ vanish in the $\varepsilon$-neighborhood of the lattice $\Gamma ^*$,

(ii) $l_c$ is the space of sequences $q(n)\in R^3$ such that $q(n)=0$, $n>N$ for some *N*.
(iii) $$ {\mathscr {D}}:=\{Y=(\Psi _1,\Psi _2,q,p): \Psi _1,\Psi _2\in {\mathscr {S}}_+,~~~ q, p\in l_c \}.$$

Obviously, ${\mathscr {D}}$ is dense in ${{\mathscr {Y}}^0}$.

### Theorem 4.2

Let conditions (1.2) and (2.13) hold. Then $B{\mathscr {D}}\subset {{\mathscr {Y}}^0}$ and *B* is a symmetric operator on the domain ${\mathscr {D}}$.

### Proof

Formally the matrix (1.15) is symmetric. The following lemma implies that *B* is defined on ${\mathscr {D}}$.$\square$

### Lemma 4.3

(i) $$H^0\Psi \in L^2({\mathbb R}^3)$$ for $$\Psi \in {\mathscr {S}}_+$$.

(ii) $\psi ^0 G\psi ^0 \Psi \in L^2({\mathbb R}^3)$ and $S^*\Psi \in l^2$ for $\Psi \in {\mathscr {S}}_+$.
(iii) $$S q\in L^2({\mathbb R}^3)$$ for $$q\in l_c$$.

### Proof

(i) $$H^0\Psi (x):=(-\frac{1}{2} \Delta -e\Phi ^0(x)-\omega ^0)\Psi (x)\in L^2({\mathbb R}^3)$$ since $$\Phi ^0\in H^2(T^3)\subset C_b({\mathbb R}^3)$$.

(ii) Given a fixed $\varphi \in {\mathscr {S}}_+$, we have $\varphi \in {\mathscr {S}}_\varepsilon$ with some $\varepsilon >0$. First, we note that
  4.1 $$\begin{aligned} G\psi ^0\Psi =F^{-1}\frac{[\tilde{\psi }^0*\tilde{\Psi }](\xi )}{|\xi |^2}, \end{aligned}$$
  where *F* stands for the Fourier transform. Further, $\tilde{\psi }^0(\xi )=(2\pi )^3\sum _{m\in {\mathbb Z}^3} \check{\psi }^0(m)\delta (\xi -2\pi m)$. Respectively,
  4.2 $$\begin{aligned}{}[\tilde{\psi }^0*\tilde{\Psi }](\xi )= (2\pi )^3\sum _{m\in {\mathbb Z}^3} \check{\psi }^0(m)\hat{\Psi }(\xi -2\pi m)=0, \qquad |\xi |<\varepsilon \end{aligned}$$
  Moreover, $\psi ^0(x)$ is a bounded function by (2.13). As a result, $\psi ^0\Psi \in L^2({\mathbb R}^3)$ and $\tilde{\psi }^0*\tilde{\Psi }\in L^2({\mathbb R}^3)$. Hence, $\Psi$ belongs to the domain of $G\psi ^0$ and of $\psi ^0G\psi ^0$. We now consider $S^*\Psi$. Applying (3.3), the Parseval identity and (4.2), we get for $\Psi \in {\mathscr {S}}_\varepsilon$
  4.3 $$\begin{aligned}{}[S^*\Psi ](n)= & {} e\int \psi ^0(x)\Psi (x)G\nabla \sigma (x-n)dx =e\langle \psi ^0(x)\Psi (x),G\nabla \sigma (x-n)\rangle \nonumber \\= & {} \frac{ie}{(2\pi )^{3}}\int _{|\xi |>\varepsilon }[\tilde{\psi }^0*\tilde{\Psi }](\xi ) \frac{\xi \overline{\tilde{\sigma }}(\xi )e^{-in\xi }}{|\xi |^2}d\xi . \end{aligned}$$
  Here $\partial ^\alpha [\tilde{\psi }^0*\tilde{\Psi }] \in L^2({\mathbb R}^3)$ for all $\alpha$ by (2.14), since $\tilde{\Psi }\in {\mathscr {S}}({\mathbb R}^3)$. Moreover, $\partial ^\alpha \tilde{\sigma }\in L^2({\mathbb R}^3)$ for $|\alpha |\le 2$ by (3.2). Hence, integrating by parts twice and taking into account (4.2), we obtain
  4.4 $$\begin{aligned} |[S^*\Psi ](n)|\le C\langle n\rangle ^{-2}, \end{aligned}$$
  which implies that $S^*\Psi \in l^2$.
(iii) Let us check that $Sq\in L^2({\mathbb R}^3)$ for $q\in l_c$. Calculating the Fourier transform of *Sq*, we obtain that
  4.5 $$\begin{aligned} \widetilde{Sq}(\xi )= & {} e F_{x\rightarrow \xi }\sum _n \psi ^0(x)G\nabla \sigma (x-n)q(n) =e\sum _n \tilde{\psi }^0*F_{x\rightarrow \xi }[G\nabla \sigma (x-n)]q(n) \nonumber \\= & {} e(2\pi )^3 \int \sum _m \check{\psi }^0(m) \delta (\eta -2\pi m) \widetilde{G\nabla \sigma }(\xi -\eta ) \sum _{n}e^{in(\xi -\eta )}q(n)d\eta \nonumber \\= & {} e(2\pi )^3 \sum _m \check{\psi }^0(m) \widetilde{G\nabla \sigma }(\xi -2\pi m) \tilde{q}(\xi -2\pi m). \end{aligned}$$
  where $\tilde{q}$ means the Fourier transform (3.6) extended $\Gamma ^*$-periodically to ${\mathbb R}^3$. Now the Parseval identity gives that
  4.6 $$\begin{aligned} \Vert Sq\Vert _{L^2({\mathbb R}^3)}=(2\pi )^{-3}\Vert \widetilde{Sq}\Vert _{L^2({\mathbb R}^3)} \le C \Vert \widetilde{G\nabla \sigma }(\xi )\tilde{q}(\xi )\Vert _{L^2({\mathbb R}^3)} \sum _m |\check{\psi }^0(m)|. \end{aligned}$$
  It remains to note that the sum over *m* is finite by (2.14), and
  4.7 $$\begin{aligned} \Vert \widetilde{G\nabla \sigma } \tilde{q}\Vert _{L^2({\mathbb R}^3)}^2= \int \frac{1}{|\xi |^2}|\tilde{\sigma }(\xi ) \tilde{q}(\xi )|^2d\xi \le C(q)\int \frac{|\tilde{\sigma }(\xi )|^2}{|\xi |^2}d\xi \end{aligned}$$
  since the function $\tilde{q}(\xi )$ is bounded for $q\in l_c$. Finally, the last integral is finite by (3.2). $\square$ This lemma implies that $BY\in {{\mathscr {Y}}^0}$ for $Y\in {\mathscr {D}}$. The symmetry of *B* on ${\mathscr {D}}$ is evident from (1.15). Theorem 4.2 is proved.$\square$

### Remark 4.4

The infrared singularity at $\xi =0$ of the integrands (4.1), (4.3) and (4.7) demonstrates that all operators $G\psi ^0:L^2({\mathbb R}^3)\rightarrow L^2({\mathbb R}^3)$, $S:l^2\rightarrow L^2({\mathbb R}^3)$ and $S^*:L^2({\mathbb R}^3)\rightarrow l^2$ are unbounded.

### Corollary 4.5

The proof of Theorem 4.2 shows that $A{\mathscr {D}}\subset {\mathscr {Y}}^0$, and also $A^*{\mathscr {D}}\subset {\mathscr {Y}}^0$, where the ‘formal adjoint’ $A^*$ is defined by the identity

4.8 $$\begin{aligned} \langle AY_1,Y_2\rangle =\langle Y_1,A^*Y_2\rangle ,\qquad Y_1,Y_2\in {\mathscr {D}}. \end{aligned}$$

## Factorization of Energy and Bound from Below

The equation (1.14) is formally a Hamiltonian system with the Hamiltonian functional $\frac{1}{2}\langle Y,B Y \rangle$.

### Theorem 5.1

Let conditions (1.2) and (2.13) hold. Then the operator *B* on the domain ${\mathscr {D}}$ is bounded from below,

5.1 $$\begin{aligned} \langle Y,BY \rangle \ge -C\Vert Y\Vert ^2_{{{\mathscr {Y}}^0}},~~~~~~~Y\in {\mathscr {D}}, \end{aligned}$$

where $$C>0$$.

### Proof

For $Y=(\Psi _1,\Psi _2,q,p)\in {\mathscr {D}}$ the quadratic form reads as

5.2 $$\begin{aligned} \langle Y,BY \rangle= & {} 2\sum _{j=1}^2\langle \Psi _j,H^0\Psi _j \rangle + 4e^2\langle \psi ^0\Psi _1,G\psi ^0\Psi _1\rangle + 2[\langle \Psi _1, S q \rangle +\langle q, S^{{\,}*} \Psi _1 \rangle ] + \langle q,T_1q\rangle \nonumber \\&+\,\langle q,T_2q\rangle + \langle p,M^{-1}p\rangle \end{aligned}$$

with the notation (3.3)–(3.4), where $\psi ^0\in C_b^2({\mathbb R}^3)$ by (2.13). Here the first sum is bounded from below, the operator $T_2$ is bounded in $l^2$ by Lemma 3.1, while the operator $M^{-1}$ is positive. Our basic observation is that

5.3 $$\begin{aligned} \beta (\Psi _1,q):= 4e^2\langle \psi ^0\Psi _1,G\psi ^0\Psi _1\rangle +2[\langle \Psi _1, S q \rangle +\langle q, S^{{\,}*} \Psi _1 \rangle ] +\langle q,T_1q\rangle \ge 0. \end{aligned}$$

Indeed, the operators factorize as follows:

5.4 $$\begin{aligned} e^2\psi ^0 G\psi ^0=f^*f, \qquad S=f^*g, \qquad T_1= g^*g; \end{aligned}$$

here

5.5 $$\begin{aligned} f:=e\sqrt{G}\psi ^0,\qquad g(x,n)={\nabla }{\sqrt{G}}\sigma (x-n). \end{aligned}$$

Now the quadratic form (5.3) becomes the ‘perfect square’

5.6 $$\begin{aligned} \beta (\Psi _1,q)= \langle 2f \Psi _1 +gq, 2f \Psi _1 +gq \rangle \ge 0. \end{aligned}$$

$$\square $$

### Corollary 5.2

The operator *B* with the domain ${\mathscr {D}}$ admits selfadjoint extensions by the Friedrichs extension theorem [26].

## Generator in the Fourier–Bloch Transform

We reduce the operators *A* and *B* with the help of the Fourier–Bloch–Gelfand–Zak transform [8, 25, 27].

### The Discrete Fourier Transform

Let us consider a vector $Y=(\Psi _1, \Psi _2, q, p)\in {{\mathscr {Y}}^0}$ and denote

6.1 $$\begin{aligned} Y(n)=(\Psi _1(n,\cdot ),\Psi _2(n,\cdot ), q(n),p(n))~,~~~~~~n\in {\mathbb Z}^3, \end{aligned}$$

where

6.2 $$\begin{aligned} \Psi _j(n,y)= \Psi _j(n+y)~~\mathrm{for\ a.e.}~~y\in \Pi ,\qquad j=1,2. \end{aligned}$$

Obviously, *Y*(*n*) with different $n\in {\mathbb Z}^3$ are orthogonal vectors in ${{\mathscr {Y}}^0}$, and besides,

6.3 $$\begin{aligned} Y=\sum _n Y(n), \end{aligned}$$

where the sum converges in ${{\mathscr {Y}}^0}$. The norms in ${{\mathscr {Y}}^0}$ and ${{\mathscr {Y}}^1}$ can be represented as

6.4 $$\begin{aligned} \Vert Y\Vert _{{\mathscr {Y}}^0}^2=\sum _{n\in {\mathbb Z}^3}\Vert Y(n)\Vert _{{{\mathscr {Y}}^0}(\Pi )}^2,\quad \quad \Vert Y\Vert _{{\mathscr {Y}}^1}^2=\sum _{n\in {\mathbb Z}^3}\Vert Y(n)\Vert _{{{\mathscr {Y}}^1}(\Pi )}^2, \end{aligned}$$

where

6.5 $$\begin{aligned} {{\mathscr {Y}}^0}(\Pi ):=L^2(\Pi )\oplus L^2(\Pi ) \oplus {\mathbb C}^3\oplus {\mathbb C}^3,\quad \quad {{\mathscr {Y}}^1}(\Pi ):=H^1(\Pi )\oplus H^1(\Pi ) \oplus {\mathbb C}^3\oplus {\mathbb C}^3.\nonumber \\ \end{aligned}$$

Further, the periodic minimizer (1.9) is invariant with respect to translations of the lattice $\Gamma$, and hence the operator *A* commutes with these translations. Namely, (3.3) implies that

6.6 $$\begin{aligned} S (x,n)=S(x-n,0), \end{aligned}$$

since $\psi ^0(x)$ is a $\Gamma$-periodic function. Similarly, (3.4) implies that *T* commutes with translations of $\Gamma$. Hence, *A* can be reduced by the discrete Fourier transform

6.7 $$\begin{aligned} \hat{Y}(\theta )=F_{n\rightarrow \theta }Y(n):=\sum \limits _{n\in {\mathbb Z}^3}e^{in\theta }Y(n) =(\hat{\Psi }_1(\theta ,\cdot ),\hat{\Psi }_2(\theta ,\cdot ),\hat{q}(\theta ),\hat{p}(\theta )) \quad ~~\mathrm{for \ a.e.}~~\theta \in {\mathbb R}^3,\nonumber \\ \end{aligned}$$

where

6.8 $$\begin{aligned} \hat{\Psi }_j(\theta ,y)=\sum \limits _{n\in {\mathbb Z}^3}e^{in\theta }\Psi _j(n+y) \quad ~~\mathrm{for \ a.e.}~~\theta \in {\mathbb R}^3,\quad ~~\mathrm{a.e.}~~y\in {\mathbb R}^3. \end{aligned}$$

The function $\hat{Y}(\theta )$ is $\Gamma ^*$-periodic in $\theta$. The series (6.7) converges in $L^2(\Pi ^*,{{\mathscr {Y}}^0}(\Pi ))$, since the series (6.3) converges in ${{\mathscr {Y}}^0}$. The inversion formula is given by

6.9 $$\begin{aligned} Y(n)=|\Pi ^*|^{-1}\int _{\Pi ^*}e^{-in\theta }\hat{Y}(\theta )d\theta \end{aligned}$$

[cf. (3.6)]. The Parseval–Plancherel identity gives

6.10 $$\begin{aligned} \Vert Y\Vert _{{\mathscr {Y}}^1}^2=|\Pi ^*|^{-1}\Vert \hat{Y}\Vert _{L^2(\Pi ^*,{{\mathscr {Y}}^1}(\Pi ))}^2, \qquad \Vert Y\Vert _{{\mathscr {Y}}^0}^2=|\Pi ^*|^{-1}\Vert \hat{Y}\Vert _{L^2(\Pi ^*,{{\mathscr {Y}}^0}(\Pi ))}^2. \end{aligned}$$

The functions $\hat{\Psi }_j(\theta ,y)$ are $\Gamma$-quasiperiodic in *y*; i.e.,

6.11 $$\begin{aligned} \hat{\Psi }_j(\theta ,y+m)=e^{-im\theta }\hat{\Psi }_j (\theta ,y), \qquad m\in {\mathbb Z}^3. \end{aligned}$$

### Generator in the Discrete Fourier Transform

Let us consider $Y\in {\mathscr {D}}$ and calculate the Fourier transform (6.7) for *AY* given by (1.14) assuming (1.2) and (2.13). Using (3.4), (4.3), (6.6), and taking into account the $\Gamma$-periodicity of $\Phi ^0(x)$ and $\psi ^0(x)$, we obtain

6.12 $$\begin{aligned} \widehat{AY}(\theta )=\hat{A}(\theta ){\hat{Y}}(\theta )\qquad ~~\mathrm{for \ a.e.}~~\theta \in {\mathbb R}^3\setminus \Gamma ^*, \end{aligned}$$

where $\hat{A}(\theta )$ is a $\Gamma ^*$-periodic operator function,

6.13 $$\begin{aligned} \hat{A}(\theta )=\left( \!\begin{array}{cccl} 0 &{}\quad H^0 &{}\quad 0 &{}\quad 0\\ -H^0-2e^2\psi ^0\hat{G}(\theta )\psi ^0 &{}\quad 0 &{}\quad \hat{S}(\theta ) &{}\quad 0\\ 0 &{}\quad 0 &{}\quad 0 &{}\quad M^{-1}\\ -2\hat{S}^{{\,}*}(\theta )&{}\quad 0 &{}\quad -\hat{T}(\theta )&{}\quad 0\\ \end{array}\!\right) \end{aligned}$$

by (1.14) and (1.15). Here

6.14 $$\begin{aligned} \hat{G}(\theta )\hat{\Psi }(\theta ,y)=\sum _m\frac{\check{\Psi }(\theta ,m)}{(2\pi m+\theta )^2} e^{-i2\pi m y}\qquad ~~\mathrm{for \ a.e.}~~ \theta \in {\mathbb R}^3\setminus \Gamma ^*. \end{aligned}$$

This expression is well-defined for $\Psi (x)=\psi ^0(x)\Psi _1(x)$ with $\Psi _1\in {\mathscr {S}}_\varepsilon$, since

6.15 $$\begin{aligned} \check{\Psi }(\theta ,m)=\tilde{\Psi }(2\pi m+\theta )=0 \quad \mathrm{for}\quad |2\pi m+\theta |<\varepsilon \end{aligned}$$

according to (4.2).

#### Lemma 6.1

Let (1.2) and (2.13) hold. Then the operator $\hat{S}(\theta )$ acts as follows:

6.16 $$\begin{aligned} \widehat{S q}(\theta ) =\hat{S}(\theta )\hat{q}(\theta ), \quad \mathrm{where}\quad \hat{S}(\theta )=e\psi ^0\hat{G}(\theta )\nabla \hat{\sigma }(\theta ,y). \end{aligned}$$

#### Proof

For $x=y+n$ equations (2.4) and (3.3) imply

$$\begin{aligned} S q(y+n)= & {} e\psi ^0(y+n)\sum _m G\nabla \sigma ^0(m,y+n)q(m)\\= & {} e\psi ^0(y)\sum _mG\nabla \sigma (y+n-m)q(m) \end{aligned}$$

due to the $\Gamma$-periodicity of $\psi ^0$. Applying the Fourier transform (6.7), we obtain (6.16).$\square$

Furthermore, $\hat{S}^{{\,}*}(\theta )$ in (6.13) is the corresponding adjoint operator, and $\hat{T}(\theta )$ is the operator matrix expressed by (3.9) and (3.12). Note that $\hat{S}(\theta )$, $\hat{S}^{{\,}*}(\theta )$ and $\hat{T}(\theta )$ are finite-rank operators.

### Generator in the Bloch Transform

#### Definition 6.2

The Bloch transform of $Y\in {{\mathscr {Y}}^0}$ is defined as

6.17 $$\begin{aligned} \tilde{Y}(\theta )=[{\mathscr {F}}Y](\theta ):= {\mathscr {M}}(\theta )\hat{Y}(\theta ): =(\tilde{\Psi }_1(\theta ,\cdot ),\tilde{\Psi }_2(\theta ,\cdot ),\hat{ q}(\theta ),\hat{ p}(\theta )) \qquad ~~\mathrm{for \ a.e.}~~\theta \in {\mathbb R}^3,\nonumber \\ \end{aligned}$$

where $\tilde{\Psi }_j(\theta ,y)=M(\theta )\hat{\Psi }_j:=e^{i\theta y}\hat{\Psi }_j(\theta ,y)$ are $\Gamma$-periodic functions in $y\in {\mathbb R}^3$.

Now the Parseval-Plancherel identities (6.10) read

6.18 $$\begin{aligned} \Vert Y\Vert _{{\mathscr {Y}}^1}^2= |\Pi ^*|^{-1}\Vert \tilde{Y}\Vert _{L^2(\Pi ^*,{{\mathscr {Y}}^1}(T^3))}^2, \qquad \Vert Y\Vert _{{\mathscr {Y}}^0}^2 = |\Pi ^*|^{-1}\Vert \tilde{Y}\Vert _{L^2(\Pi ^*,{{\mathscr {Y}}^0}(T^3))}^2. \end{aligned}$$

Hence, ${\mathscr {F}}:{{\mathscr {Y}}^0}\rightarrow L^2(\Pi ^*,{{\mathscr {Y}}^0}(T^3))$ is an isomorphism. The inversion is given by

6.19 $$\begin{aligned} Y(n)=|\Pi ^*|^{-1}\int _{\Pi ^*}e^{-in\theta }{\mathscr {M}}(-\theta )\tilde{Y}(\theta )d\theta ,\qquad n\in {\mathbb Z}^3. \end{aligned}$$

Finally, the above calculations can be summarised as follows: (6.12) implies that, for $Y\in {\mathscr {D}}$,

6.20 $$\begin{aligned} \widetilde{AY}(\theta )= \tilde{A}(\theta ) \tilde{Y}(\theta )\quad ~~\mathrm{for \ a.e.}~~\theta \in \Pi ^*\setminus \Gamma ^*. \end{aligned}$$

Here,

6.21 $$\begin{aligned} \tilde{A}(\theta )\!=\!{\mathscr {M}}(\theta )\hat{A}(\theta ){\mathscr {M}}(-\theta ) \!=\!\!\left( \!\!\! \begin{array}{cccl} 0 &{}\quad \!\!\tilde{H}^0(\theta ) &{}\quad 0 &{}\quad 0\\ -\tilde{H}^0(\theta )-2e^2\psi ^0\tilde{G}(\theta )\psi ^0&{}\quad 0 &{}\quad ~\tilde{S}(\theta ) &{}\quad 0\\ 0 &{}\quad 0&{}\quad \!0&{}\quad \!M^{-1}\\ -2\tilde{S}^{{\,}*}(\theta )\!\!&{}\quad 0 &{}\quad -\hat{T}(\theta )&{}\quad \!0\\ \end{array}\!\!\!\!\right) ,\nonumber \\ \end{aligned}$$

where

6.22 $$\begin{aligned} \tilde{S}(\theta ):= & {} M(\theta )\hat{S}(\theta )= e\psi ^0\tilde{G}(\theta )\nabla \tilde{\sigma }^0(\theta ),\end{aligned}$$

6.23 $$\begin{aligned} \tilde{H}^0(\theta ):= & {} M(\theta ) H^0M(-\theta )=-\frac{1}{2}(\nabla -i\theta )^2-e\Phi ^0(x)-\omega ^0,\end{aligned}$$

6.24 $$\begin{aligned} \tilde{G}(\theta ):= & {} M(\theta ) \hat{G}(\theta ) M(-\theta )=(i\nabla +\theta )^{-2}. \end{aligned}$$

Formula (6.20) is obtained for $Y\in {\mathscr {D}}$. Respectively, the operator (6.21) is considered on the space ${\mathscr {D}}(T^3):=C^\infty (T^3)\oplus C^\infty (T^3)\oplus {\mathbb C}^3\oplus {\mathbb C}^3$ up to now. However, $\Phi ^0\in H^2(T^3)\subset C_b({\mathbb R}^3)$ and $\psi ^0\in C^2_b({\mathbb R}^3)$ by (2.13). Hence, the operator (6.21) extends uniquely to the continuous operator ${\mathscr {Y}}^2(T^3)\rightarrow {\mathscr {Y}}^0(T^3)$ for $\theta \in \Pi ^*\setminus \Gamma ^*$. We keep below the notation (6.21)–(6.24) for this extension.

#### Remark 6.3

The operators $\tilde{G}(\theta ):L^2(T^3)\rightarrow H^2(T^3)$ are bounded for $\theta \in \Pi ^*\setminus \Gamma ^*$; however $\Vert \tilde{G}(\theta )\Vert \sim d^{-2}(\theta )$, where $d(\theta ):=\mathrm dist{\,}(\theta ,\Gamma ^*)$.

#### Lemma 6.4

Let conditions (1.2) and (2.13) hold. Then the operator $\tilde{A}(\theta )$ admits the representation

6.25 $$\begin{aligned} \tilde{A}(\theta )=J\tilde{B}(\theta ),~~~~~~~~~~\theta \in \Pi ^*\setminus \Gamma ^*, \end{aligned}$$

where $\tilde{B}(\theta )$ is the selfadjoint operator (1.17) in ${{\mathscr {Y}}^0}(T^3)$ with the domain ${{\mathscr {Y}}^2}(T^3)$.

#### Proof

The representation (6.25) follows from (1.15). The operator $\tilde{B}(\theta )$ is symmetric on the domain ${\mathscr {Y}}^2(T^3)$. Moreover, all operators in (1.17), except for $\tilde{H}^0(\theta )$, are bounded. Finally, $\tilde{H}^0(\theta )$ is selfadjoint in $L^2(T^3)$ with the domain $H^2(T^3)$. Hence, $\tilde{B}(\theta )$ is also selfadjoint on the domain ${\mathscr {Y}}^2(T^3)$. $\square$

## The Positivity of Energy

Here we prove the positivity (1.20) under conditions (1.21) and (1.23). In this case the real periodic minimizer is given by (1.24) with $\phi =0$, and hence,

7.1 $$\begin{aligned} \hat{T}(\theta )=\hat{T}_1(\theta )=\Sigma (\theta ),\qquad \theta \in \Pi ^*\setminus \Gamma ^*. \end{aligned}$$

by Lemma 3.1(ii) and (3.9).

It is easy to construct examples of densities $\sigma (x)$ satisfying conditions (1.21) and (1.23).

### Example 7.1

(1.21) holds for $\sigma$ satisfying (1.2) if

7.2 $$\begin{aligned} \tilde{\sigma }(\xi )\ne 0\qquad ~~\mathrm{for \ a.e.}~~\xi \in {\mathbb R}^3. \end{aligned}$$

### Example 7.2

Let us define the function *s*(*x*) by its Fourier transform $\tilde{s}(\xi ):=\displaystyle \frac{2\sin \displaystyle \frac{\xi }{2}}{\xi }e^{-\xi ^2}$, and set

7.3 $$\begin{aligned} \sigma (x):=eZ s(x_1)s(x_2)s(x_3),\qquad x\in {\mathbb R}^3. \end{aligned}$$

Then $\sigma (x)$ is a holomorphic function of $x\in {\mathbb C}^3$ satisfying conditions (1.21), (1.23), (1.1), (1.2 ), and besides,

7.4 $$\begin{aligned} |\partial ^\alpha \sigma (x)|\le C(a,\alpha )e^{-a|x|},~~~~x\in {\mathbb R}^3, \end{aligned}$$

for any $a>0$ and $\alpha$ by the Paley–Wiener theorem.

The matrix (1.21) is a continuous function of $\theta \in \Pi ^*\setminus \Gamma ^*$. Let us denote

7.5 $$\begin{aligned} \Pi ^*_+:=\{\theta \in \Pi ^*\setminus \Gamma ^*: \Sigma (\theta )>0 \}. \end{aligned}$$

Then the Wiener condition (1.21) means that $|\Pi ^*_+|=|\Pi ^*|$. Let us recall that we consider the energy operator $\tilde{B}(\theta )$ corresponding to the real periodic minimizer (1.24) with $\phi =0$. The main result of present paper is the following theorem.

### Theorem 7.3

Let conditions (1.23) and (1.2) hold. Then

(i) The Wiener condition (1.21) is necessary and sufficient for the positivity (1.20), and the bound (1.26) holds.
(ii) Bound (1.25) holds with sufficiently small $\varepsilon >0$ under the Wiener condition (1.21).

### Proof

(i) First, let us check that the Wiener condition (1.21) is necessary. Namely, let us consider inequality (1.20) for $\tilde{Y}=(0,0,\hat{q},0)\in {{\mathscr {Y}}^1}(T^3)$. Using (1.17), this gives
  7.6 $$\begin{aligned} {\mathscr {E}}(\theta ,\tilde{Y}) =\hat{q}\hat{T}(\theta )\hat{q} \ge \varkappa (\theta )|\hat{q}|^2\quad ~~\mathrm{for \ a.e.}~~\theta \in \Pi ^*\setminus \Gamma ^*. \end{aligned}$$
  Now (7.1) gives
  7.7 $$\begin{aligned} {\mathscr {E}}(\theta ,\tilde{Y}) = \hat{q}\Sigma (\theta )\hat{q} \ge \varkappa (\theta )|\hat{q}|^2. \end{aligned}$$
  Hence, the condition (1.21) is necessary for the positivity (1.20). Moreover, (7.7) implies (1.26).
(ii) It remains to show that the Wiener condition (1.21) together with (1.23) is sufficient for the bound (1.25). Let us translate the calculations (5.2)–(5.5) into the Fourier–Bloch transform. The operators (5.5) commute with the $\Gamma$-translations, and therefore
  7.8 $$\begin{aligned} e^2\psi ^0\tilde{G}(\theta )\psi ^0=\tilde{f}^*(\theta )\tilde{f}(\theta ), \quad \tilde{S}(\theta )=\tilde{f}^*(\theta )\tilde{g}(\theta ), \quad \hat{T}_1(\theta )= \tilde{g}^*(\theta )\tilde{g}(\theta ), \end{aligned}$$
  where $\tilde{f}(\theta ):=e\sqrt{\tilde{G}(\theta )}\psi ^0$ and $\tilde{g}(\theta )={\sqrt{\tilde{G}(\theta )}}\nabla \tilde{\sigma }(\cdot ,\theta )$. Hence, (1.17) implies that
  7.9 $$\begin{aligned} {\mathscr {E}}(\theta ,\tilde{Y}):= \langle \tilde{Y},\tilde{B}(\theta )\tilde{Y} \rangle _{{{\mathscr {Y}}^0}(T^3)}\!=\!b(\theta , \tilde{\Psi }_1,\hat{q})+ 2\langle \tilde{\Psi }_2,\tilde{H}^0(\theta ) \tilde{\Psi }_2 \rangle _{L^2(T^3)} + \hat{p}M^{-1} \hat{p}\nonumber \\ \end{aligned}$$
  for $$\tilde{Y}=(\tilde{\Psi }_1, \tilde{\Psi }_2, \hat{q}, \hat{p})\in {\mathscr {Y}}^2(T^3)$$, where
  7.10 $$\begin{aligned} b(\theta , \tilde{\Psi }_1,\hat{q}) := 2\langle \tilde{\Psi }_1,\tilde{H}^0(\theta ) \tilde{\Psi }_1 \rangle _{L^2(T^3)}+ \langle 2\tilde{f}(\theta ) \tilde{\Psi }_1 + \tilde{g}(\theta ) \hat{q}, ~2\tilde{f}(\theta ) \tilde{\Psi }_1 +\tilde{g}(\theta ) \hat{q} \rangle _{L^2(T^3)}.\nonumber \\ \end{aligned}$$
  Let us note that $\tilde{H}^0(\theta )= -\displaystyle \frac{1}{2}(\nabla +i\theta )^2$ by (1.24). Hence, the eigenvalues of $\tilde{H}^0(\theta )$ equal to $\displaystyle \frac{1}{2}|2\pi m-\theta |^2$, where $m\in {\mathbb Z}^3$. Therefore, $\tilde{H}^0(\theta )$ is positive definite: for $j=1,2$
  7.11 $$\begin{aligned} \langle \tilde{\Psi }_j, \tilde{H}^0(\theta )\tilde{\Psi }_j \rangle \ge \frac{1}{2} d^2(\theta )\Vert \tilde{\Psi }_j\Vert _{H^1(T^3)}^2~, \qquad \theta \in \Pi ^*\setminus \Gamma ^*. \end{aligned}$$
  Hence, it remains to prove the following lemma, since $\Sigma _0(\theta )>0$ for $\theta \in \Pi _+^*$ by (1.21).

### Lemma 7.4

Under conditions of Theorem 7.3 for any $\theta \in \Pi ^*_+$ there exists $\varepsilon _1>0$ such that

7.12 $$\begin{aligned} b(\theta , \tilde{\Psi }_1,\hat{q}) \ge \frac{1}{2} d^2(\theta )\Vert \tilde{\Psi }_1\Vert _{H^1(T^3)}^2+\varepsilon _1 d^4(\theta ) \Sigma _0(\theta )|\hat{q}|^2. \end{aligned}$$

### Proof

Let us denote7.13Then we can write the quadratic form (7.10) as

7.14 $$\begin{aligned} b=2\alpha +\beta , \end{aligned}$$

where $$\alpha :=\langle \tilde{\Psi }_1,\tilde{H}^0(\theta ) \tilde{\Psi }_1 \rangle _{L^2(T^3)}\ge 0$$ and

7.15 $$\begin{aligned} \beta := \beta _{11}+2\mathrm{Re{\,}}\beta _{12}+\beta _{22}= \langle 2\tilde{f}(\theta ) \tilde{\Psi }_1 + \tilde{g}(\theta ) \hat{q}, ~2\tilde{f}(\theta ) \tilde{\Psi }_1+\tilde{g}(\theta ) \hat{q} \rangle _{L^2(T^3)} \ge 0.\nonumber \\ \end{aligned}$$

By (7.11) it suffices to prove the estimate

7.16 $$\begin{aligned} b\ge \alpha +\varepsilon d^4(\theta ) \beta _{22}, \end{aligned}$$

since

7.17 $$\begin{aligned} \beta _{22}= \hat{q}\hat{T}_1(\theta )\hat{q}= \hat{q}\Sigma (\theta )\hat{q} \end{aligned}$$

by (7.8) and (7.1). To prove (7.16), we first note that

7.18 $$\begin{aligned} \alpha \ge \varepsilon _2d^4(\theta ) \beta _{11},\qquad \theta \in \Pi ^*\setminus \Gamma ^*, \end{aligned}$$

where $\varepsilon _2>0$ by (7.11). Indeed, (6.24) and (1.24) imply that

7.19 $$\begin{aligned} \beta _{11}=4\langle \tilde{\Psi }_1, \tilde{G}(\theta )\tilde{\Psi }_1\rangle \le \frac{C}{d^2(\theta )}\Vert \tilde{\Psi }_1\Vert ^2_{L^2(T^3)}, \end{aligned}$$

and moreover, $d^2(\theta )\Vert \tilde{\Psi }_1\Vert _{L^2(T^3)}^2\le \langle \tilde{\Psi }_1,(i\nabla +\theta )^2 \tilde{\Psi }_1 \rangle _{L^2(T^3)}=2\alpha$ by (6.23).

Now (7.18) and (7.14) give that

7.20 $$\begin{aligned} b\ge \alpha + (1+ \varepsilon _2 d^4(\theta )) \beta _{11}+2\mathrm{Re{\,}}\beta _{12}+\beta _{22},\qquad \theta \in \Pi ^*\setminus \Gamma ^*. \end{aligned}$$

On the other hand, the Cauchy–Schwarz inequality implies that

7.21 $$\begin{aligned} |\beta _{12}|\le \beta _{11}^{1/2}\beta _{22}^{1/2}\le \frac{1}{2}[\gamma \beta _{11}+\frac{1}{\gamma }\beta _{22}] \end{aligned}$$

for any $\gamma >0$. Hence, (7.20) implies that

7.22 $$\begin{aligned} b\ge \alpha + (1+ \varepsilon _2 d^4(\theta )-\gamma ) \beta _{11}+(1-\frac{1}{\gamma })\beta _{22},\qquad \theta \in \Pi ^*\setminus \Gamma ^*. \end{aligned}$$

Choosing $\gamma = 1+ \varepsilon _2 d^4(\theta )$, we obtain (7.16). $\square$

At last, formula (7.9) and estimates (7.11), (7.12) imply (1.25) with sufficiently small $\varepsilon >0$.$\square$

### Corollary 7.5

Bound (1.25) implies that (1.19) holds with

7.23 $$\begin{aligned} \inf _{\theta \in K}\varkappa (\theta )>0 \end{aligned}$$

for any compact subset $K\subset \Pi ^*_+$.

### Remark 7.6

Lemma 7.4 and its proof were inspired by the Sylvester criterion for the positivity of $2\times 2$ matrices. Namely, in notation (7.13) for the matrix $\beta =(\beta _{ij})$ we have $\beta _{11}\ge 0$, $\beta _{22}> 0$. Furthermore, the matrix $\beta \ge 0$, since it corresponds to the perfect square, and hence ${\text {det}} \beta \ge 0$. Therefore, the Sylvester criterion implies that

7.24 $$\begin{aligned} \beta _+:=\left( \begin{array}{cc} \alpha +\beta _{11}&{}\quad \beta _{12}\\ \beta _{21}&{}\quad \beta _{22} \end{array} \right) >0 \end{aligned}$$

since $\alpha +\beta _{11}> 0$, $\beta _{22}> 0$ and $\det \beta _+=\alpha \beta _{22}+\det \beta >0$. These arguments are behind our estimates (7.20)–(7.22), which give (7.16).

## Weak Solutions and Linear Stability

We introduce weak solutions and prove the linear stability of the dynamics (1.14) assuming (1.2), (1.21) and (1.23). Then the real periodic minimizer is given by (1.24) with $\phi =0$, and (1.19) and (1.25) hold by Theorem 7.3.

### Weak Solutions 

Let us define solutions $Y(t)\in C({\mathbb R},{\mathscr {Y}}^1)$ to (1.14) in the sense of vector-valued distributions of $t\in {\mathbb R}$. Let us recall that $A^*V\in {{\mathscr {Y}}^0}$ for $V\in {\mathscr {D}}$ by Corollary . We call *Y*(*t*) a weak solution to (1.14) if, for every $V\in {\mathscr {D}}$,

8.1 $$\begin{aligned} \langle Y(t)-Y(0),V\rangle = \int _0^t\langle Y(s),A^*V \rangle ds,\qquad t\in {\mathbb R}. \end{aligned}$$

Equivalently, by the Parseval–Plancherel identity,

8.2 $$\begin{aligned} \int _{\Pi ^*}\langle \tilde{Y}(\theta ,t)-\tilde{Y}(\theta ,0),\tilde{V}(\theta )\rangle _{{{\mathscr {Y}}^0}(T^3)} d\theta = \int _0^t\Big [\int _{\Pi ^*} \langle \tilde{Y}(\theta ,s),\tilde{A}^*(\theta )\tilde{V}(\theta ) \rangle _{{{\mathscr {Y}}^0}(T^3)} d\theta \Big ]ds\nonumber \\ \end{aligned}$$

Fubini’s theorem implies that

8.3 $$\begin{aligned} \tilde{Y}(\theta ,\cdot )\in L^1_\mathrm{loc} ({\mathbb R},{\mathscr {Y}}^1(T^3))\qquad \text{ for } \text{ a.e. }\quad \theta \in \Pi ^*, \end{aligned}$$

and (8.2) is equivalent to

8.4 $$\begin{aligned} \int _{\Pi ^*}\langle \tilde{Y}(\theta ,t)-\tilde{Y}(\theta ,0),\tilde{V}(\theta )\rangle _{{{\mathscr {Y}}^0}(T^3)} d\theta = \int _{\Pi ^*}\Big [\int _0^t \langle \tilde{Y}(\theta ,s),\tilde{A}^*(\theta )\tilde{V}(\theta ) \rangle _{{{\mathscr {Y}}^0}(T^3)}ds\Big ] d\theta .\nonumber \\ \end{aligned}$$

Equivalently,

8.5 $$\begin{aligned} \langle \tilde{Y}(\theta , t)-\tilde{Y}(\theta , 0),\tilde{V}\rangle _{{{\mathscr {Y}}^0}(T^3)} \!=\! \int _0^t \langle \tilde{Y}(\theta ,s),\tilde{A}^*(\theta )\tilde{V} \rangle _{{{\mathscr {Y}}^0}(T^3)} ds,\quad t\in {\mathbb R},\quad ~~ \tilde{V}\!\in \! {\mathscr {D}}(T^3)\nonumber \\ \end{aligned}$$

for a.e. $$\theta \in \Pi ^*\setminus \Gamma ^*$$. Formally,

8.6 $$\begin{aligned} \dot{\tilde{Y}}(\theta , t)= \tilde{A}(\theta ) \tilde{Y}(\theta ,t),\qquad t\in {\mathbb R}\end{aligned}$$

for a.e. $\theta \in \Pi ^*\setminus \Gamma ^*$ in the sense of vector-valued distributions.

### Reduction to Mild Solution

We reduce (8.6) to an equation with a selfadjoint generator by using (1.19) and our methods [17, 18]. By (1.19) and (1.25) the operator $\tilde{\Lambda }(\theta ):=\tilde{B}^{1/2}(\theta )>0$ is invertible in ${{\mathscr {Y}}^0}(T^3)$ for $\theta \in \Pi ^*_+$ and

8.7 $$\begin{aligned} \Vert \tilde{\Lambda }^{-1}(\theta )Z\Vert _{{{\mathscr {Y}}^1}(T^3)}\le \frac{1}{\sqrt{ \varkappa (\theta )}} \Vert Z\Vert _{{{\mathscr {Y}}^0}(T^3)}, \quad Z\in {{\mathscr {Y}}^0}(T^3),\qquad \theta \in \Pi ^*_+. \end{aligned}$$

Hence, $\tilde{A}(\theta )=J\tilde{B}(\theta )$ and $\tilde{A}^*(\theta )=-\tilde{B}(\theta )J$ are also invertible in ${{\mathscr {Y}}^0}(T^3)$. Therefore, (8.5) can be rewritten as

8.8 $$\begin{aligned} \langle \tilde{Y}(\theta , t)-\tilde{Y}(\theta , 0),(\tilde{A}^*(\theta ))^{-1}\tilde{W}\rangle _{{{\mathscr {Y}}^0}(T^3)}= & {} \int _0^t \langle \tilde{Y}(\theta ,s),\tilde{W} \rangle _{{{\mathscr {Y}}^0}(T^3)} ds,\qquad t\in {\mathbb R},\nonumber \\&\tilde{W}\!\in \! \tilde{A}^*(\theta ){\mathscr {D}}(T^3) \end{aligned}$$

for a.e. $$\theta \in \Pi ^*_+$$.

#### Lemma 8.1

The linear space $\tilde{A}^*(\theta ){\mathscr {D}}(T^3)$ is dense in ${{\mathscr {Y}}^0}(T^3)$.

#### Proof

First, $\tilde{A}^*(\theta ){\mathscr {D}}(T^3)=\tilde{B}(\theta ){\mathscr {D}}(T^3)$, since $J{\mathscr {D}}(T^3)={\mathscr {D}}(T^3)$. Second, $\tilde{B}(\theta )$, which is defined on ${\mathscr {D}}(T^3)$, extends to an invertible selfadjoint operator in ${{\mathscr {Y}}^0}(T^3)$ with the domain ${{\mathscr {Y}}^2}(T^3)$ and $\mathrm{Ran}\tilde{B}(\theta )={{\mathscr {Y}}^0}(T^3)$.$\square$

As a corollary, (8.8) is equivalent to the ‘mild solution’ identity

8.9 $$\begin{aligned} \tilde{A}^{-1}(\theta ) [\tilde{Y}(\theta , t)-\tilde{Y}(\theta , 0)] =\! \int _0^t \tilde{Y}(\theta ,s) ds,\qquad t\in {\mathbb R}\qquad \text{ for } \text{ a.e. } \theta \in \Pi ^*_+. \end{aligned}$$

### Reduction to Selfadjoint Generator

Now we can apply our approach [17] to reduce (8.9) to the dynamics with a selfadjoint generator. By (8.3)

8.10 $$\begin{aligned} Z(\theta ,\cdot ):=\tilde{\Lambda }(\theta )\tilde{Y}(\theta ,\cdot )\in L^1_\mathrm{loc} ({\mathbb R},{\mathscr {Y}}^0(T^3)) \qquad \text{ for } \text{ a.e. }\quad \theta \in \Pi ^*. \end{aligned}$$

Hence, applying $\tilde{\Lambda }(\theta )$ to the both sides of (8.9), we obtain the equivalent equation

8.11 $$\begin{aligned} \tilde{K}^{-1}(\theta ) [\tilde{Z}(\theta , t)-\tilde{Z}(\theta , 0)] =-i \int _0^t \tilde{Z}(\theta ,s) ds,\qquad t\in {\mathbb R}\qquad \text{ for } \text{ a.e. }\quad \theta \in \Pi ^*, \end{aligned}$$

where $\tilde{K}(\theta ):=i\tilde{\Lambda }(\theta ) J\tilde{\Lambda }(\theta )$, since $\tilde{A}^{-1}(\theta )=\tilde{\Lambda }^{-2}(\theta )J^{-1}$. Formally,

8.12 $$\begin{aligned} \dot{\tilde{Z}}(\theta ,t)=-i\tilde{K}(\theta )\tilde{Z}(\theta ,t),\quad t\in {\mathbb R}\qquad \text{ for } \text{ a.e. }\quad \theta \in \Pi ^* \end{aligned}$$

in the sense of vector-valued distributions.

Now the problem is that the domain of $\tilde{K}(\theta )$ is unknown since the ion density $\sigma (x)$ generally is not smooth, so we cannot use the PDO techniques. The following lemma plays a key role in our approach (cf. Lemma 2.1 of [17]).

#### Lemma 8.2

(i) $\tilde{K}(\theta )$ is a selfadjoint operator in ${{\mathscr {Y}}^0}(T^3)$ with a dense domain $D_\theta =D(\tilde{K}(\theta ))\subset {{\mathscr {Y}}^1}(T^3)$ for every $\theta \in \Pi ^*_+$.
(ii) The eigenvectors of $\tilde{K}(\theta )$ form a complete set in ${{\mathscr {Y}}^0}(T^3)$.

#### Proof

(i) The operator $\tilde{K}(\theta )$ is injective. On the other hand, $\mathrm{Ran}{\,}\tilde{\Lambda }(\theta )={{\mathscr {Y}}^0}(T^3)$, and $J:{{\mathscr {Y}}^0}(T^3)\rightarrow {{\mathscr {Y}}^0}(T^3)$ is a bounded invertible operator. Hence, $\mathrm{Ran}{\,}\tilde{K}(\theta )={{\mathscr {Y}}^0}(T^3)$. Consider the inverse operator
  8.13 $$\begin{aligned} \tilde{R}(\theta ):=\tilde{K}^{-1}(\theta )=i\tilde{\Lambda }^{-1}(\theta ) J^{-1}\tilde{\Lambda }^{-1}(\theta ). \end{aligned}$$
  This operator is selfadjoint, since it is bounded and symmetric. Hence, $\mathrm{Ran}{\,} \tilde{K}(\theta )=D(\tilde{R}(\theta ))={{\mathscr {Y}}^0}(T^3)$. Therefore, $\tilde{K}(\theta )=\tilde{R}^{-1}(\theta )$ is a densely defined selfadjoint operator by Theorem 13.11, (b) of [28]:
  $$\begin{aligned} \tilde{K}^*(\theta )=\tilde{K}(\theta )~, \quad D(\tilde{K}(\theta ))=\mathrm{Ran}{\,} \tilde{R}(\theta )\subset \ \mathrm{Ran}{\,}\tilde{\Lambda }^{-1}(\theta )\subset {{\mathscr {Y}}^1}(T^3) \end{aligned}$$
  where the last inclusion follows by (8.7).
(ii) (8.7) implies that $\tilde{\Lambda }^{-1}(\theta )$ is a compact operator in ${{\mathscr {Y}}^0}(T^3)$ by the Sobolev embedding theorem. Hence, $\tilde{K}^{-1}(\theta )$ is also compact operator in ${{\mathscr {Y}}^0}(T^3)$ by (8.13).

$$\square $$

This lemma implies that the formula

8.14 $$\begin{aligned} \tilde{Z}(\theta ,t)=e^{-i \tilde{K}(\theta ) t}\tilde{Z}(\theta ,0) \in C_b({\mathbb R},{{\mathscr {Y}}^0}(T^3)) \end{aligned}$$

gives a unique solution to (8.12) for each $\theta \in \Pi ^*_+$ and every $\tilde{Z}(\theta ,0)\in {{\mathscr {Y}}^0}(T^3)$. Indeed, it suffices to expand $Z(\theta ,t)$ in the eigenvectors of $\tilde{K}(\theta )$ and to note that (8.11) gives ordinary differential equations for each component. Now we can prove the well posedness of the Cauchy problem for equation (8.6) with any $\theta \in \Pi ^*_+$.

#### Theorem 8.3

Let conditions (1.21), (1.23) and (1.2) hold, the periodic minimizer $\psi ^0$ is given by (1.24) with $\phi =0$, and $\theta \in \Pi ^*_+$. Then, for every initial state $\tilde{Y}(\theta ,0)\in {{\mathscr {Y}}^1}(T^3)$, there exists a unique solution $\tilde{Y}(\theta ,\cdot )\in C_b({\mathbb R},{{\mathscr {Y}}^1}(T^3))$ to equation (8.6) in the sense of (8.5). Besides,

8.15 $$\begin{aligned} \langle \tilde{\Lambda }(\theta ) \tilde{Y}(\theta ,t), \tilde{\Lambda }(\theta )\tilde{Y}(\theta ,t)\rangle _{{{\mathscr {Y}}^0}(T^3)}=C(\theta ), \qquad t\in {\mathbb R}. \end{aligned}$$

#### Proof

First, we note that $\tilde{Z}(\theta ,0):=\tilde{\Lambda }(\theta )\tilde{Y}(\theta ,0)\in {{\mathscr {Y}}^0}(T^3)$. Hence, (8.14) and (8.7) imply that

8.16 $$\begin{aligned} \tilde{Y}(\theta ,t)=\tilde{\Lambda }^{-1}(\theta ) e^{-i K(\theta ) t} \tilde{Z}(\theta ,0) \in C_b({\mathbb R},{{\mathscr {Y}}^1}(T^3)) \end{aligned}$$

is the unique solution to (8.6). Finally,

$$\begin{aligned} \langle \tilde{\Lambda }(\theta )\tilde{Y}(\theta ,t),\tilde{\Lambda }(\theta )\tilde{Y}(\theta ,t)\rangle _{{{\mathscr {Y}}^0}(T^3)}= \langle \tilde{Z}(\theta ,t),\tilde{Z}(\theta ,t)\rangle _{{{\mathscr {Y}}^0}(T^3)}=C(\theta ),\qquad t\in {\mathbb R}, \end{aligned}$$

since $e^{-i K(\theta ) t}$ is the unitary group in ${{\mathscr {Y}}^0}(T^3)$. $\square$

### Linear Stability in the Energy Space

Thus, we have constructed $\tilde{Y}(\theta ,t)$ uniquely for a.e. $\theta \in \Pi ^*_+$. However, (8.15) does not imply that there exists the corresponding $Y(t)\in {\mathscr {Y}}^1$, since $\tilde{\Lambda }(\theta )$ can degenerate at some points $\theta \in \Pi ^*\setminus \Pi ^*_+$. In particular, it degenerates at $\theta =0$ due to (1.26) and (1.27). Thus, we need another phase space to construct solutions to (8.1). Let us denote

$$\begin{aligned} {\mathscr {D}}_0:=\{Y\in {{\mathscr {Y}}^1}: \tilde{Y}(\theta )=0 ~~\text{ in } \text{ a } \text{ neighborhood } \text{ of }~~\Gamma ^*\}. \end{aligned}$$

Lemma 6.4 implies that $\tilde{\Lambda }(\theta )\tilde{Y}(\theta )\in L^2(\Pi ^*_+,{{\mathscr {Y}}^0}(T^3))$ for $Y\in {\mathscr {D}}_0$. Moreover, Theorem 7.3 shows that

8.17 $$\begin{aligned} \Vert Y \Vert _{\mathscr {W}}:=\Vert \tilde{\Lambda }(\theta )\tilde{Y}(\theta ) \Vert _{L^2(\Pi ^*_+,{{\mathscr {Y}}^0}(T^3))}> 0,\qquad Y\in {\mathscr {D}}_0\setminus 0 \end{aligned}$$

under conditions (1.21), (1.23) and (1.2). Hence, $\Vert Y \Vert _{\mathscr {W}}$ is a norm on ${\mathscr {D}}_0$.

#### Definition 8.4

The Hilbert space ${\mathscr {W}}$ is the completion of ${\mathscr {D}}_0$ in the norm $\Vert Y \Vert _{\mathscr {W}}$.

Formally, we have $\Vert Y\Vert _{\mathscr {W}}=\langle Y,BY\rangle ^{1/2}$. By Corollary 7.5, the Fourier–Bloch transform (6.17) extends to the isomorphism

8.18 $$\begin{aligned} {\mathscr {F}}:{\mathscr {W}}\rightarrow \tilde{\mathscr {W}}:=\{\tilde{Y}(\cdot )\in L^2_\mathrm{loc}(\Pi ^*_+,{{\mathscr {Y}}^0}(T^3)): \Vert \tilde{\Lambda }(\theta )\tilde{Y}(\theta ) \Vert _{L^2(\Pi ^*_+,{{\mathscr {Y}}^0}(T^3))}<\infty \}.\qquad \end{aligned}$$

Hence, we can extend the definition of weak solutions (8.1) to $Y(t)\in C({\mathbb R},{\mathscr {W}})$ by identity (8.1) with $V\in {\mathscr {D}}$ such that

8.19 $$\begin{aligned} {\text {supp}}\tilde{V}(\theta )\subset \Pi ^*_+. \end{aligned}$$

Theorem 8.3 has the following corollary, which is one of main results of present paper.

#### Corollary 8.5

Let all conditions of Theorem 8.3 hold. Then, for every initial state $Y(0)\in {\mathscr {W}}$, there exists a unique weak solution $Y(\cdot )\in C_b({\mathbb R},{\mathscr {W}})$ to Eq. (1.14), the energy norm being conserved:

8.20 $$\begin{aligned} \Vert Y(t)\Vert _{\mathscr {W}}=\mathop {\mathrm {const}}\nolimits ,\qquad t\in {\mathbb R}. \end{aligned}$$

The solution is given by formula (8.16):

8.21 $$\begin{aligned} Y(t)={\mathscr {F}}^{-1}\tilde{\Lambda }^{-1}(\theta ) e^{-i K(\theta ) t} \tilde{\Lambda }(\theta )\tilde{Y}(\theta ,0) \in C_b({\mathbb R},{\mathscr {W}}). \end{aligned}$$

The energy conservation (8.20) follows from (8.15) by integration over $\theta \in \Pi ^*_+$.

This means that the linearized dynamics (1.14) is stable in the ‘energy space’ ${\mathscr {W}}$: a global solution exists and is unique for each initial state of finite energy, and the ‘energy norm’ is constant in time.

## Small-Charge Asymptotics of the Periodic Minimizer

We will need below the asymptotics as $e\rightarrow 0$ of the periodic minimizer (1.9) corresponding to a one-parametric family of ion densities

9.1 $$\begin{aligned} \sigma (x)=e\mu (x) \end{aligned}$$

with some fixed function $\mu \in L^2({\mathbb R}^3)$. We assume that

9.2 $$\begin{aligned} \mu ^0(x):=\sum _{n\in {\mathbb Z}^3} \mu (x-n)\in L^2(T^3) \end{aligned}$$

in accordance with (2.7). Now the energy (2.8) reads

9.3 $$\begin{aligned} U(\psi )=\frac{1}{2}\int _{T^3}[|\nabla \psi (x)|^2 +e^2\nu (x)G_\mathrm{per}\nu (x)]dx,\qquad \nu (x):=\mu ^0(x)-|\psi (x)|^2. \end{aligned}$$

Denote by $\psi ^0_e,\omega ^0_e$ the family of periodic minimizers with the parameter $e\in (0,1]$. Formulas (1.24) do not hold in general, since we do not assume (1.23).

The energy (9.3) is obviously bounded uniformly in $e\in (0,1]$ for any fixed $\psi \in {\mathscr {M}}$. Hence, the energy of the minimizers is also bounded uniformly in $e\in (0,1]$. In particular, the family $\psi ^0_e$ is bounded in $H^1(T^3)$, and hence in $L^6(T^3)$ by the Sobolev embedding theorem,

9.4 $$\begin{aligned} \Vert \psi ^0_e \Vert _{H^1(T^3)}+\Vert \psi ^0_e \Vert _{L^6(T^3)}\le C, \qquad e\in (0,1]. \end{aligned}$$

Therefore,

9.5 $$\begin{aligned} \int \nu ^0_e(x)G_\mathrm{per}\nu ^0_e(x)dx\le C ,\qquad \nu ^0_e(x):= \mu ^0(x)-|\psi _e^0(x)|^2. \end{aligned}$$

This estimate follows from the uniform bound

9.6 $$\begin{aligned} \big \Vert \nu ^0_e\big \Vert _{L^2(T^3)}\le C,\qquad e\in (0,1] \end{aligned}$$

which holds by (9.2), (9.4) and (2.10). Further, the equation (2.2) reads

9.7 $$\begin{aligned} -\Delta \Phi ^0_e(x)=e\nu ^0_e(x). \end{aligned}$$

We will choose the solution $\Phi ^0_e=eG_\mathrm{per}\nu ^0_e$, where the operator $G_\mathrm{per}$ is defined by (2.10). Then

9.8 $$\begin{aligned} \big \Vert \Phi ^0_e\big \Vert _{H^2(T^3)}\le e\Vert \nu ^0_e\Vert _{L^2(T^3)} \le Ce, \qquad e\in (0,1] \end{aligned}$$

by (9.6).

### Lemma 9.1

Let condition (9.2) hold. Then the periodic minimizer admits the following asymptotics as $e\rightarrow 0$:

9.9 $$\begin{aligned} \omega ^0_e={\mathscr {O}}(e^2), \end{aligned}$$

9.10 $$\begin{aligned} \psi ^0_e(x)=\gamma _e+\chi _e(x),\qquad |\gamma _e|^2=Z+{\mathscr {O}}(e^4), \qquad \Vert \chi _e\Vert _{H^2(T^3)}= {\mathscr {O}}(e^2). \end{aligned}$$

### Proof

(i) Equation (2.1) reads as
  9.11 $$\begin{aligned} \omega ^0_e\psi ^0_e(x)=-\frac{1}{2}\Delta \psi ^0_e(x)-e\Phi ^0_e(x)\psi ^0_e(x),\qquad x\in T^3. \end{aligned}$$
  Hence,
  9.12 $$\begin{aligned} \omega ^0_e\langle \psi ^0_e,\psi ^0_e\rangle _{L^2(T^3)}=\omega ^0_e Z= \frac{1}{2}\langle \nabla \psi ^0_e, \nabla \psi ^0_e\rangle _{L^2(T^3)}-e\langle \Phi ^0_e\psi ^0_e,\psi ^0_e\rangle _{L^2(T^3)}, \end{aligned}$$
  which implies the uniform bound
  9.13 $$\begin{aligned} |\omega ^0_e|\le C<\infty ,\qquad e\in (0,1] \end{aligned}$$
  by (2.6), (9.4) and (9.8). Moreover, (9.11) and (9.8) suggest that $\omega ^0_e$ is close to an eigenvalue of $-\frac{1}{2}\Delta$:
  9.14 $$\begin{aligned} \omega ^0_e\approx |2\pi k|^2 \end{aligned}$$
  with some $k\in {\mathbb Z}^3$. Indeed, (9.11) can be rewritten as
  9.15 $$\begin{aligned} \left( \frac{1}{2} |2\pi m|^2-\omega ^0_e\right) \check{\psi }^0_e(m)= \check{r_e}(m),\qquad r_e:=e\Phi ^0_e\psi ^0_e \end{aligned}$$
  and hence,
  9.16 $$\begin{aligned} \sum _{m\in {\mathbb Z}^3} \left( \frac{1}{2} |2\pi m|^2-\omega ^0_e\right) ^2|\check{\psi }^0_e(m)|^2={\mathscr {O}}(e^4), \end{aligned}$$
  since $\Vert r_e\Vert _{L^2(T^3)}={\mathscr {O}}(e^2)$ by (9.8). Denote by $\lambda _e$ the value of $|2\pi m|^2$ corresponding to the minimal magnitude of $|\frac{1}{2} |2\pi m|^2-\omega ^0_e|$. Now (9.16) implies that
  9.17 $$\begin{aligned} \sum _{|2\pi m|^2\ne \lambda _e} |\check{\psi }^0_e(m)|^2 ={\mathscr {O}}(e^4), \end{aligned}$$
  since the set of possible values of $\frac{1}{2}|2\pi m|^2-\omega ^0_e$ is discrete and the possible values of $\omega ^0_e$ are bounded by (9.13). Moreover, (9.16) can be rewritten as
  9.18 $$\begin{aligned} \left( \frac{1}{2}\lambda _e-\omega ^0_e\right) ^2Z +\sum _{|2\pi m|^2\ne \lambda _e}\Big [\left( \frac{1}{2} |2\pi m|^2-\omega ^0_e\right) ^2-\left( \frac{1}{2}\lambda _e-\omega ^0_e\right) ^2\Big ] |\check{\psi }^0_e(m)|^2 ={\mathscr {O}}(e^4),\nonumber \\ \end{aligned}$$
  since
  9.19 $$\begin{aligned} \sum _{m\in {\mathbb Z}^3} |\check{\psi }^0_e(m)|^2= Z \end{aligned}$$
  due to the normalization (2.6). Hence,
  9.20 $$\begin{aligned} \left| \frac{1}{2}\lambda _e-\omega ^0_e\right| ={\mathscr {O}}(e^2), \end{aligned}$$
  since the sum in (9.18) is nonnegative.

(ii) Let us show that (9.18) also implies that
  9.21 $$\begin{aligned} \sum _{|2\pi m|^2\ne \lambda _e}(|2\pi m|^2-\lambda _e)^2|\check{\psi }^0_e(m)|^2={\mathscr {O}}(e^4). \end{aligned}$$
  First, (9.18) gives that
  $$\begin{aligned} \sum _{|2\pi m|^2\ne \lambda _e} (|2\pi m|^2-\lambda _e)\left( \frac{1}{2} |2\pi m|^2+\frac{1}{2}\lambda _e-2\omega ^0_e\right) |\check{\psi }^0_e(m)|^2 ={\mathscr {O}}(e^4). \end{aligned}$$
  However, $2\omega ^0_e=\lambda _e+{\mathscr {O}}(e^2)$ by (9.20). Hence,
  $$\begin{aligned} \sum _{|2\pi m|^2\ne \lambda _e} (|2\pi m|^2-\lambda _e)(|2\pi m|^2-\lambda _e+{\mathscr {O}}(e^2)) |\check{\psi }^0_e(m)|^2 ={\mathscr {O}}(e^4). \end{aligned}$$
  Now (9.21) follows from (9.17), since $\lambda _e$ is bounded for small $e>0$ by (9.20) and (9.13).
(iii) Now let us prove that $\lambda _e=0$ for small $e>0$. Indeed, the energy of the periodic minimizer reads
  9.22 $$\begin{aligned} U(\psi ^0_e)=\frac{1}{2}\sum _{m\in {\mathbb Z}^3} |2\pi m|^2|\check{\psi }^0_e(m)|^2+{\mathscr {O}}(e^2) \end{aligned}$$
  by (9.3) and (9.5). On the other hand, (9.21) implies that
  9.23 $$\begin{aligned} \sum _m |2\pi m|^2|\check{\psi }^0_e(m)|^2 =\lambda _e Z+\sum _{|2\pi m|^2\ne \lambda _e} (|2\pi m|^2-\lambda _e)|\check{\psi }^0_e(m)|^2 =\lambda _e Z+{\mathscr {O}}(e^4).\nonumber \\ \end{aligned}$$
  Substituting (9.23) into (9.22), we obtain
  9.24 $$\begin{aligned} U(\psi ^0_e) =\frac{1}{2}\lambda _e Z+{\mathscr {O}}(e^2),\quad \lambda _e\ge 0. \end{aligned}$$
  On the other hand, taking $\psi (x)\equiv \sqrt{Z}$, we ensure that the energy minimum (2.11) does not exceed ${\mathscr {O}}(e^2)$. Hence, (9.24) implies that $\lambda _e=0$ for small $e>0$, since the set of all possible values of $\lambda _e Z$ is discrete. Therefore, (9.9) holds by (9.20).
iv) Now we can prove the asymptotics (9.10). Namely, the first identity holds if we set
  9.25 $$\begin{aligned} \gamma _e=\check{\psi }^0_e(0),\qquad \chi _e(x)= \sum _{m\ne 0} e^{-i2\pi mx}\check{\psi }^0_e(m). \end{aligned}$$
  Then the second asymptotics of (9.10) holds by (9.19) and (9.17) with $\lambda _e=0$. The last asymptotics of (9.10) holds, since
  9.26 $$\begin{aligned} \sum _{m\ne 0} |2\pi m|^4|\check{\psi }^0_e(m)|^2={\mathscr {O}}(e^4) \end{aligned}$$
  due to (9.21) with $\lambda _e=0$.

$$\square $$

## Examples of Negative Energy

We show that the positivity (1.20) can fail if the condition (1.23) breaks down even when the Wiener condition (1.21) holds. Namely, for $Y_0=(0,0,q,0)\in {{\mathscr {Y}}^1}(T^3)$ we have

10.1 $$\begin{aligned} {\mathscr {E}}(\theta ,Y_0) =q\hat{T}(\theta )q \end{aligned}$$

by (7.6).

### Lemma 10.1

There exist functions $\mu (x)$ such that the positivity (1.20) fails for $\sigma (x)$ given by (9.1) with small $e>0$, while both (1.2) and the Wiener condition (1.21) hold.

### Proof

Now formula (1.17) for $\tilde{B}(\theta )$ should be slightly modified, since we do not know wether the periodic minimizer $\psi ^0(x)$ is real up to a factor. Namely, for complex $\psi ^0(x)$ we have

10.2 $$\begin{aligned} \tilde{B}(\theta ) =\!\left( \!\begin{array}{cccc} 2\tilde{H}^0(\theta )+4e^2\psi ^0_{1} \tilde{G}(\theta )\psi ^0_{1}&{}\quad 4e^2\psi ^0_{1} \tilde{G}(\theta )\psi ^0_{2}&{}\quad 2\tilde{S}_1(\theta )&{}\quad 0 \\ 4e^2\psi ^0_{2} \tilde{G}(\theta )\psi ^0_{1}&{}\quad 2\tilde{H}^0(\theta )+4e^2\psi ^0_{2} \tilde{G}(\theta )\psi ^0_{2}&{}\quad 2\tilde{S}_2(\theta )&{}\quad 0 \\ 2\tilde{S}_1^{{\,}*}(\theta ) &{}\quad 2\tilde{S}_2^{{\,}*}(\theta ) &{} \quad \hat{T}(\theta ) &{}\quad 0 \\ 0 &{}\quad 0 &{}\quad 0 &{}\quad M^{-1} \\ \end{array}\!\right) ,\nonumber \\ \end{aligned}$$

where $\psi ^0_1(x):=\mathrm{Re{\,}}\psi ^0(x)$ and $\psi ^0_2(x):=\mathrm{Im{\,}}\psi ^0(x)$, while $\tilde{S}_1(\theta )$, $\tilde{S}_2(\theta )$ are suitable generalizations of $\tilde{S}(\theta )$. It suffices to construct an example of $\sigma (x)$ which provides

10.3 $$\begin{aligned} \hat{q}\hat{T}(\theta _0)\hat{q}<0 \end{aligned}$$

for some $\theta _0\in \Pi ^*\setminus \Gamma ^*$ and $\hat{q}\in {\mathbb C}^3$. The representation (3.9) can be written as

10.4 $$\begin{aligned} \hat{T}_1(\theta ) = e^2 \sum _m \Big [\frac{\xi \otimes \xi }{|\xi |^2}|\tilde{\mu }(\xi )|^2\Big ]_{\xi =2\pi m-\theta }~, \quad \theta \in \Pi ^*\setminus \Gamma ^*. \end{aligned}$$

Similarly, (3.12) can be written in the Fourier representation as

10.5 $$\begin{aligned} \hat{T}_2 =-e^2\frac{1}{(2\pi )^3}\langle \tilde{\nu }^0_e(\xi ) \frac{\xi \otimes \xi }{|\xi |^2}, \tilde{\mu }(\xi )\rangle \end{aligned}$$

with $\nu ^0_e:= \mu ^0(x)-|\psi _e^0(x)|^2$ according to (9.5). The asymptotics (9.10) of the periodic minimizer $\psi ^0_e(x)$ implies that

10.6 $$\begin{aligned} \tilde{\nu }^0_e(\xi )= \tilde{\mu }^0(\xi )-|\gamma _e|^2(2\pi )^3\delta (\xi )-\tilde{s}(\xi )= \tilde{\mu }^0(\xi )-Z(2\pi )^3\delta (\xi )-\tilde{s}(\xi ), \end{aligned}$$

since $|\gamma _e|^2=Z$ by (9.10). Here, $s(x)=\gamma _e\overline{\chi }_e(x)+\overline{\gamma }_e\chi _e(x)+|\chi _e(x)|^2$, and so

10.7 $$\begin{aligned} \Vert s\Vert _{L^2(T^3)}\le C_1 e^2 \end{aligned}$$

by (9.10). Further, (9.2) gives

10.8 $$\begin{aligned} \tilde{\mu }^0(\xi )=\sum _n\tilde{\mu }(\xi )e^{in\xi }= \tilde{\mu }(\xi )(2\pi )^3\sum _m\delta (\xi -2\pi m) \end{aligned}$$

by the Poisson summation formula [15]. Substituting (10.8) into (10.6) we get

10.9 $$\begin{aligned} \tilde{\nu }^0_e(\xi ) =\tilde{\mu }(\xi )(2\pi )^3\sum _{m\ne 0} \delta (\xi -2\pi m)-\tilde{s}(\xi ) \end{aligned}$$

by (1.1) and (9.1). Substituting this expression into (3.12) we obtain

10.10 $$\begin{aligned} \hat{T}_2= -e^2\langle \tilde{\mu }(\xi ) \sum _{m\ne 0} \delta (\xi -2\pi m) \frac{\xi \otimes \xi }{|\xi |^2}, \tilde{\mu }(\xi )\rangle + \frac{e^2}{(2\pi )^3}\langle \tilde{s}(\xi ) \frac{\xi \otimes \xi }{|\xi |^2}, \tilde{\mu }(\xi )\rangle .\qquad \quad \end{aligned}$$

Further, *s*(*x*) is a $\Gamma$-periodic function, and now,

$$\begin{aligned} \int _{T^3} s(x)dx=\int _{T^3} \nu ^0_e(x)dx=0 \end{aligned}$$

by (9.7). Hence,

10.11 $$\begin{aligned} \tilde{s}(\xi )=\sum _{m\ne 0} \check{s}(m)\delta (\xi -2\pi m),\qquad \sum _m|\check{s}(m)|^2={\mathscr {O}}(e^4),\qquad e\rightarrow 0 \end{aligned}$$

by (10.7). Therefore,

10.12 $$\begin{aligned} \hat{T}_2= -e^2\sum _{m\ne 0 } \Big [\frac{\xi \otimes \xi }{|\xi |^2}|\tilde{\mu }(\xi )|^2\Big ]_{\xi =2\pi m}+{\mathscr {O}}(e^4),\qquad e\rightarrow 0. \end{aligned}$$

Hence, there exists a $\hat{q}\in {\mathbb C}^3$ such that

10.13 $$\begin{aligned} \hat{q}\hat{T}_2\hat{q}<0 \end{aligned}$$

for small $e>0$ if the condition (1.23) breaks down. For example, we can take $\hat{q}=2\pi m$ with $m\in {\mathbb Z}^3\setminus 0$ if $\tilde{\mu }(2\pi m)\ne 0$. Finally, for any $\theta _0\not \in \Gamma ^*$ we can reduce $|\hat{\mu }(\theta )|$ at all points $\theta \in \theta _0+\Gamma ^*$ keeping it at all points of $\Gamma ^*$ to have

10.14 $$\begin{aligned} \hat{q}\hat{T}(\theta _0)\hat{q}=\hat{q}\hat{T}_1(\theta _0)\hat{q}+\hat{q}\hat{T}_2\hat{q}<0. \end{aligned}$$

At the same time, we can keep (1.2) and the Wiener condition (1.21) to hold.$\square$

### Remark 10.2

The operator $T_2$ corresponds to the last term in the last line of (1.11). This term describes the ‘virtual repulsion’ of the ion located around the nod *n* from the same ion deflected to the point $n+q(n,t)$. This means that the negative energy contribution is provided by the electrostatic instability (‘Earnshaw’s Theorem’ [29]).

## Appendix 1: Formal Linearization at the Periodic Minimizer

Let us substitute

$$\begin{aligned} \psi (x,t)=[\psi ^0(x)+\Psi (x,t)]e^{-i\omega ^0 t} \end{aligned}$$

into the nonlinear equations (1.3), (1.5) with $\Phi (x,t)=G\rho (x,t)$. First, (1.4) implies that

$$\begin{aligned} \rho (x,t)=\sum _n\sigma (x-n-q (n,t))-e|\psi ^0(x)+\Psi (x,t)|^2 \end{aligned}$$

and the Taylor expansion *formally* gives

11.1 $$\begin{aligned}&\rho (x,t) = \sum _n\Big [\sigma (x-n)-\nabla \sigma (x-n) q(n,t)+ \frac{1}{2}\nabla \nabla \sigma (x-n)q(n,t)\otimes q(n,t)+\cdots \Big ] \nonumber \\&\quad -\, e\Big [|\psi ^0(x)|^2+2\mathrm{Re{\,}}(\psi ^0(x)\overline{\Psi }(x,t))+|\Psi (x,t)|^2\Big ] =\rho ^0(x)+\rho _1(x,t)+\rho _2(x,t)+\cdots \nonumber \\ \end{aligned}$$

Here $\rho ^0(x):=\sigma ^0(x)-e|\psi ^0(x)|^2$ and $\rho _k$ are polynomials in $\Psi (x,t)$ and *q*(*t*) of degree *k*. In particular, $\rho _1(x,t)$ is given by (1.12). As a result, we obtain the system (1.11) in the linear approximation.
